# No molecular or serological evidence of Zikavirus infection among healthy blood donors living in or travelling to regions where Aedes *albopictus* circulates

**DOI:** 10.1371/journal.pone.0178175

**Published:** 2017-05-24

**Authors:** Wegene Borena, Tamara Hofer, Karin Stiasny, Stephan W. Aberle, Manfred Gaber, Dorothee von Laer, Harald Schennach

**Affiliations:** 1 Division of Virology, Medical University of Innsbruck, Innsbruck, Austria; 2 Center for Virology, Medical University of Vienna, Vienna, Austria; 3 Tyrolean Red Cross blood donor service, Innsbruck, Austria; 4 Central Institute for Blood Transfusion and Immunology, Medical University Hospital Innsbruck, Innsbruck, Austria; International Centre for Genetic Engineering and Biotechnology, ITALY

## Abstract

**Background:**

Previous studies have shown that Zika virus can infect and be transmitted by A. *albopictus*. The World Health organization (WHO) has raised concerns of autochthonous transmission of the virus in regions where the vector is endemic. The aim of this pilot study was to assess the occurrence of Zika virus (ZIKV) in western Austria (Tyrol) especially after a history of travel to A. *albopictus* endemic regions.

**Methods:**

The study participants were healthy blood donors at randomly selected donation sites in the west Austrian region Tyrol. Rest blood (plasma) samples were tested for the presence of ZIKV nucleic acid and antibodies against the virus.

**Results:**

Mean age of the study participants was 44.6 (SD = 12.9) and 58.8% were men. Eighty percent reported to have received vaccine against TBEV, whereas only 4.9 and 0.9% had received YFV and JEV vaccines. Three out of 1001 (0.03%) participants tested positive solely for ZIKV IgM antibody but not for other flaviviruses. Only one individual had ZIKV IgG antibody. All four donors were negative in the neutralization (confirmation) assay. No viral RNA was detected in any of the samples.

**Conclusion:**

The null finding of our study refutes WHO’s initial fear of global expansion of ZIKV infection including its occurrence in Europe. There appears to be no urgent need to introduce universal screening of donated blood for ZIKV in central Europe at least until the next warm season. Further, Euroimmun anti-Zika ELISA proved to be a highly suitable and reliable test system in populations with high prevalence of TBEV infection and/or immunization.

## Introduction

Due to the devastating complications of fetal anomalies and autoimmune mediated peripheral polyneuropathy following ZIKV infection, due to its rapid expansion in the affected regions and due to the potential for global occurrence, ZIKV infection was declared by the World Health Organisation (WHO) as “Public Health Emergency of International Concern”[1–2].

Although the species Aedes *aegypti*–endemic in tropical and sub-tropical climates–is the main vector known to transmit ZIKV infection, other Aedes mosquito-species, also endemic in Europe–for example *A*. *albopictus* (Asian tiger mosquito)–were confirmed to carry and transmit the virus [3–4]. Similar to Dengue Virus (DEV) and Chikungunya Virus (CHIKV) [5], this may translate into possible autochthonous ZIKV infection in several *A*. *albopictus* endemic Mediterranean countries [6] which–at the same time–are popular destinations for summer holidays. Furthermore, the recent mosquito map of the European Center for Disease Control (ECDC) reveals expansion of the vector northwards invading countries in central and northern Europe (S1 Fig).

The summer Olympics event in Brazil with over 200 participating countries was feared to be the source of ZIKV introduction into unaffected regions [7–9]. It had been speculated that with the onset of spring and summer the increasing mosquito activities might put Europe at risk [10].

Based on this theoretical possibility of introduction of the virus and its autochthonous transmission via *A*. *albopictus*, we aimed to examine a collective of blood-donating healthy individuals for serological or molecular evidence of contact with the virus. Since there is concrete evidence that ZIKV may spread through blood transfusions [11], the results of nucleic acid amplification test (NAAT) may shed light on the necessity of routine screening of donated blood for ZIKV in the region.

## Materials and methods

### Study population

This pilot study was conducted among healthy adults aged 18–65 who donated blood at thirteen randomly selected donation-sites in the West Austrian federal state of Tyrol during the time period of August to November 2016. Participants provided written informed consent before they filled out a short questionnaire and gave a 5 mL EDTA-blood sample. The questionnaire provided information on history of travel to Latin America or Asia within the past eighteen months and/or a history of travel to established A. *albopictus* endemic areas in Europe in the time period between April to October 2016 before blood donation. Additionally, information on vaccination for tick-borne encephalitis virus (TBEV), Yellow fever (YFV) as well as Japanese encephalitis viruses (JEV) was obtained. History of known previous infection with any of the structurally relevant flaviviruses, namely TBE, YFV, JEV, West Nile Virus (WNV) and Dengue fever Virus (DEV) was also captured. The questionnaires (in German and English) are provided as supplementary materials (S1 File, S2 File).

### Anti-ZIKV ELISA

ZIKV specific immunoglobulins were detected using Anti-Zika Virus ELISA (IgG / IgM) according to the recommendations of the manufacturer (EUROIMMUN, Medizinische Labordiagnostika, Germany) [12–13]. Diluted patient samples (1:101) were incubated in microplate wells coated with highly purified ZIKV non-structural protein (NS1). We conducted separate analyses for IgM and IgG antibodies using separate secondary anti-human antibodies directed at human IgM and IgG, respectively. In order to improve the specificity of IgM antibodies, samples were pre-incubated with buffer containing rheumatoid factor absorbent. The secondary anti-human antibodies are peroxidase-labelled and lead to color reactions in case of bound anti-Zika antibodies despite thorough washing. Optical density (OD) was measured as described elsewhere [13] using BEP III system (Siemens Healthcare, Munich, Germany). Results were given semiquantitatively as corrected OD by calculating a ratio of the OD of the patient sample to the OD of calibrator (provided in the test kit). Results were interpreted as recommended by the manufacturer: positive if the OD ≥1.1, negative if OD <0.8 and borderline if OD ≥0.8 to <1.1.

In order to rule out possibly cross reacting antibodies, serum samples which tested positive for ZIKV antibodies, were additionally tested for TBEV (Enzygnost Anti-FSME-Virus IgG/IgM, Siemens), DEV, WNV and JEV (in-house ELISA tests developed at the Arbovirus reference center, Vienna) antibodies. All anti-ZIKV IgM antibody positive samples were additionally tested for Epstein-Barr-Virus (EBV) (EBV IgM Architect i2000S2, Abbott) in order to exclude positivity due to polyclonal B-cell stimulation.

### Neutralization test (NT)

ZIKV NT was performed essentially as described previously for West Nile virus at the National Arbovirus Reference Center in Vienna (Medical University of Vienna, Center for Virology) [14]. Briefly, serial dilutions of samples were mixed with 30–60 TCID50 Zika virus (strain H/PF 2013) [15], and incubated for one hour at 37°C. Vero cells were added and incubated further for 3 to 4 days. The presence of infectious (non-neutralized) virus in the supernatant was assessed by microscopic evaluation of cytopathic effects. Lack of cytopathic effect at titers ≥20 was interpreted as positive indicating the presence of specific neutralizing antibodies preventing the infection of Vero cells.

### Nucleic acid amplification test (NAAT)

ZIKV nucleic acid extraction was conducted in a fully automated manner according to the manufacturers instruction (NucliSense® easyMAG®, Biomerieux France). 200 μl EDTA-plasma was eluted into 110μl of purified RNA. For the amplification process, we used the recommended volume of 10μl of RNA extract with 20μl primer-enzyme-mix provided by the test kit to make a final reaction volume of 30 μl (RealStar® Zika Virus RT-PCR Kit 1.0 Altona Diagnostics, Germany) [16]. The test is based on real-time technology with reverse transcription of target RNA (NS1 gene region) to cDNA followed by amplification of target cDNA with simultaneous detection of amplified product using fluorescence labelled probes. The analytical sensitivity of the test l0.61 copies/μl of nucleic acid extracts 95% CI: (0.39–1.27 copies/μl). The kit is equipped with a system that controls for the quality of extracted sample (internal control) as well as positive and negative controls. The sample is considered negative, if there is no fluorescence signal detected up to the fortieth amplification cycle.

### Ethics

This study was approved by the Ethical Committee of the Medical University of Innsbruck (AN2016-0113 363/4.3).

## Results

A total of 1001 blood donors with a mean age of 44.6 (SD = 12.9) participated in the study. 41.2% of these were women. Table 1 presents baseline data of the study participants as well as relevant travel and immunization history. A great majority of the donors (80%) claimed to be vaccinated against TBEV. Of the known A. *albopictus* endemic regions, Italy was the most frequented country (60%), followed by Croatia and Greece as a destination for summer holidays.

**Table 1:** Baseline characteristics, immunization status and travel history of the study participants.

	Total (n = 1001)
**Age, years**	
Mean (SD)	44.61 (12.9)
**Sex, n (%)**	
Men	585 (58.4)
Women	414 (41.4)
Immunisation status*, n (%)	
TBEV	802 (80.1)
YFV	49 (4.9)
JEV	9 (0.9)
**Known history of illness with other flaviviruses (TBEV, YFV, JEV, DEV, WNV)**	0 (0)
**Travel history to the Americas**^§,^^**^	32 (3.2)
**Travel history to Asia**^**§**^	38 (3.8)
**Travel history to Mediterranean countries**^**§§**^	576 (7.5)
Italy	363 (36.3)
Croatia	114 (11.4)
Greece	52 (5.2)
Spain	61 (6.1)
France	34 (3.4)
Other destinations***	31(3.1)

* to have ever received a single dose of a vaccine or a booster §in the preceding 18 months before blood donation, missing data for 8 respondents

§§ during the time period of April to October 2016

** Latin America, Caribbean and Florida, missing data for 2 respondents

***most frequented other destination is Turkey

TBEV = Tick-borne encephalitis virus

YFV = Yellow fever virus

JEV = Japanse encephalitis virus

DEV = Dengue fever virus

WNV = West nile virus

**Fig 1:**
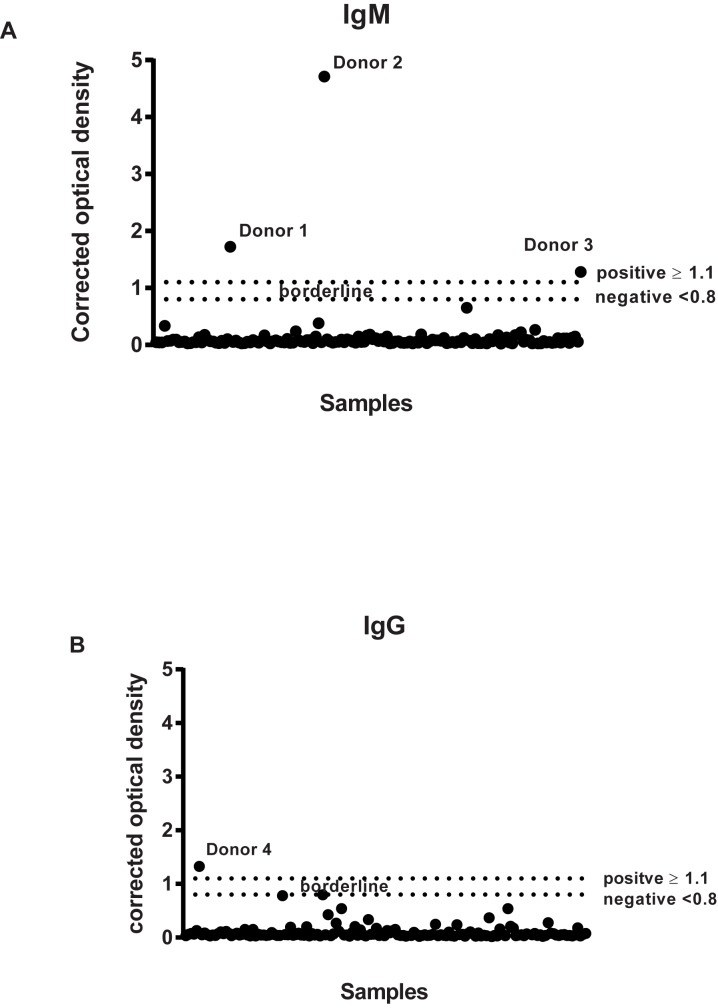
**Corrected optical density of anti-ZIKV IgM ELISA (A) and IgG ELISA (B) antibodies.** Data is shown for positive donors (Donors 1–3) (A) and (Donor 4) (B) and all samples tested in the same run for each ELISA. Corrected optical density (OD) = OD of the sample/OD of the calibrator. The test is positive if the corrected OD lies on or above the cutoff 1.1. IgM: Immunoglobulin M, IgG: immunoglobulin G, ZIKV: Zika virus.

Three individuals (0.3%) had isolated anti-ZIKV-IgM antibodies (Fig 1A), which did not react with EBV and all other structurally related flaviviruses. All three samples were negative in the neutralization assay. Convalescent sera—obtained only for one of the three participants (donor 2)–showed no IgG seroconversion. Only one participant, vaccinated against TBEV, had a positive anti-ZIKV-IgG signal (Fig 1B). ZIKV NT was negative among all ELISA positive patients failing to confirm the ZIKV-specificity of the antibodies detected.

All the samples were negative for NAAT. All the positive controls were positive; the negative controls negative and internal controls confirmed the adequacy and validity of the samples tested (S2 Fig).

## Discussion

ZIKV has been isolated from a variety of mosquito species other than A. *aegypti* including A. *albopictus*, known to be invasive, and rapidly spreading in the Mediterranean basin [17]. Despite the low transmission rate observed in experimental settings [18], this may still justify the concern that the virus could possibly be transmitted in Europe once introduced to the region as was the case with DEV and CHIKV [5]. On these grounds, the WHO gave explicit warning for individuals living and travelling to these A. *albopictus* endemic regions [8]. Although about 41% of the blood donors in our study had no history of travel to any of those Mediterranean countries [6], latest information from vector-borne disease experts (VectorNet) shows that A. *albopictus* is currently expanding northwards and has also been detected in western Austria (study region) (S1 Fig) [19].

### The issue of blood safety

The major challenge in preventing transfusion-associated transmission of ZIKV is the high rate of asymptomatic infections. Previous studies on screening of donated blood for viral RNA in ZIKV-affected regions show a prevalence of 2–3%, which is non-negligible [20–22]. With a sample size of approximately 1000 healthy donors, our study was powered to detect ZIKV prevalences as low as 0.4–1.6% and hence excludes with 95% confidence ZIKV viremia among donors in an extent observed in ZIKV endemic regions.

### ZIKV seroprevalence

Since viremia following ZIKV acquisition is limited to 5–7 days in the infection life cycle, a mere absence of ZIKV nucleic acid in blood samples does not by far rule out existence of the virus and its local transmission. Therefore, in addition to NAAT we searched for serological evidence of ZIKV activity. Although our study region is particularly known for high immunization coverage (over 80%) against TBEV [23], only one out of over 800 (0.13%) TBEV vaccinated donors was anti-Zika IgG positive confirming the high suitability of this ELISA kit in regions with high TBEV prevalence or high immunization coverage. With only 0.3% false positive IgM antibodies, which were negative for all other structurally related flaviviruses, it may be recommendable–at least in central Europe–to adopt Euroimmun ELISA as a stand-alone serological method in an acute and convalescent phase sera without necessarily going through a time consuming neutralization assay.

### Strengths and limitations of the study

Our study has strengths and some limitations. A major strength is the fact that we searched for both serological and molecular evidence of ZIKV infection using highly sensitive and validated test kits (CE marked). The availability of data on history of vaccination against and/or history of infection with other related flaviviruses made our data more valuable in terms of understanding the role of antibody cross reactivity–particularly with that of TBEV against which a great majority of the population possesses antibodies. A major limitation of the study is the lack of convalescent sera from ELISA positive samples (except for one), which would have significance in supporting or excluding a recent infection through seroconversion or rise in antibody titer. However, this diagnostic gap is narrowed to a certain extent through concomitant testing of these samples for the presence of nucleic acid and for specific neutralizing antibodies. The modest sample size, which rendered the study less powered in detecting isolated sporadic cases of ZIKV in the region, may also be a limitation. Another pitfall of the study may be the subjectivity of microscopic detection of cytopathic effects in the NT assay, which may be probe to some degree of bias. However, the complete lack of neutralizing antibodies coupled with the fact that our ELISA did not cross react with TBEV makes the interpretation of the NT assay in our study rather unequivocal.

### Conclusion

Looking into data from previous studies in endemic regions, which estimated ZIKV prevalence to reach up to 73% [24] once introduced in the area, our finding of no serological and molecular evidence among the study population may be a true reflection of the absence of autochthonous ZIKV transmission in the region. This, combined with the end of the warm season in the continent that leads to attenuation of mosquito activities, may suggest the end of ZIKV threat for now and end of the public health concern in Europe. With this result, we may dare to claim that routine screening of donated blood samples for ZIKV is most probably not a major priority in the study region. This, however, does not warrant the complete cessation of monitoring and surveillance of the virus particularly in A. *albopictus* endemic regions due to its disastrous complications once introduced. We recommend that efforts should be made to conduct similar studies in a larger scale in order to detect or exclude possible isolated sporadic occurrences.

## Supporting information

S1 Fig Regions with established and introduced A. *albopictus* (A). West Austria (Tyrol) (circled) (B) presented as a region with newly introduced A. *albopictus* (B). *(Map adapted from European center for Diease Prevention and Conrol (Mosquito Maps) Updated on October 2016*.*)*.
(TIF)

S2 Fig Diagram depicting a single run of a nucleic acid amplification test (NAAT) with 96 samples. Fluorescence signals observed for the positive control and the internal controls of each sample making the run a valid one.
(TIF)

S1 File Survey questionnaire in the German (original) language. (PDF)

S2 File Survey questionnaire in English. (PDF)

S3 File Results of ELISA photometry. (PDF)

S1 Table A raw data on country/exact destination visited by the study participants between April and October 2016. (PDF)
