# Supplementary figures and images for: No molecular or serological evidence of Zikavirus infection among healthy blood donors living in or travelling to regions where Aedes albopictus circulates
Source: PLoS One. 2017 May 24;12(5):e0178175. doi: 10.1371/journal.pone.0178175 (PMC5443526; doi:10.1371/journal.pone.0178175)

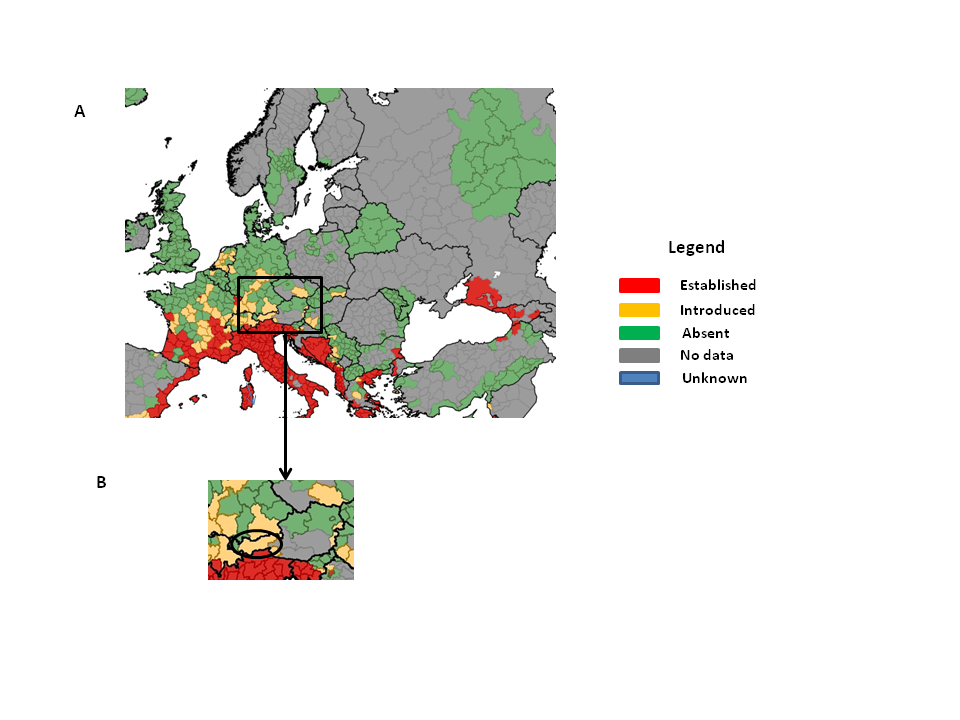

Supplement: S1 Fig — Regions with established and introduced A. albopictus (A). West Austria (Tyrol) (circled) (B) presented as a region with newly introduced A. albopictus (B). (Map adapted from European center for Diease Prevention and Conrol (Mosquito Maps) Updated on October 2016.). (TIF) [file pone.0178175.s001.tif]

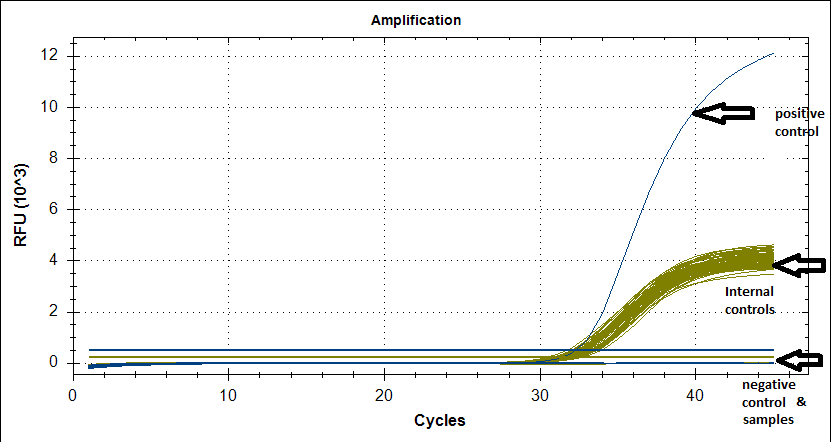

Supplement: S2 Fig — Fluorescence signals observed for the positive control and the internal controls of each sample making the run a valid one. (TIF) [file pone.0178175.s002.tif]
