# Supplementary material for: No molecular or serological evidence of Zikavirus infection among healthy blood donors living in or travelling to regions where Aedes albopictus circulates
Source: PLoS One. 2017 May 24;12(5):e0178175. doi: 10.1371/journal.pone.0178175 (PMC5443526; doi:10.1371/journal.pone.0178175)
Supplement: S1 File — (PDF) [file pone.0178175.s003.pdf]

Kode: |__|__|__|__|

Untersuchung des Vorkommens von Zikavirus-Infektionen in Tirol
(Vom Probanden/ von der Probandin auszufüllen)

Datum der Blutabnahme:

|__|__|-|__|__|-|__|__|__|__|

T T M M J J J J

---

1. Ich bin:
   ☐ Männlich ☐ Weiblich

---

2. Ich bin:
   ………… Jahre alt

---

3. Waren Sie in den letzten 18 Monaten in einem süd-/ mittel-amerikanischen Land oder in der Karibik?
   ☐ Ja ☐ Nein

   Wenn ja, wann? …………………………………………………………………….

---

4. Waren Sie in den letzten 18 Monaten in einem ostasiatischen oder südostasiatischen Land?
   ☐ Ja ☐ Nein

   Wenn ja, wann? …………………………………………………………………….

---

5. Waren Sie in den Monaten April bis Oktober 2016 in einem der folgenden mediterranen Ländern: Italien, Frankreich, Spanien, Griechenland, Kroatien, Slowenien, Mazedonien, Montenegro und Türkei?
   ☐ Ja ☐ Nein

   Wenn ja, wann? ……………………………………………….

   Wenn ja, an welchem Ort (oder Insel) genau? ………………………………………

---

6. Wurden Sie jemals gegen folgende Erkrankungen geimpft/aufgefrischt?
   ☐ FSME

☐ Ja ☐ Nein
Wenn ja, wann? ………………………………………………

☐ Gelbfieber
☐ Ja ☐ Nein
Wenn ja, wann? ………………………………………………

☐ Japanische Enzephalitis
☐ Ja ☐ Nein
Wenn ja, wann? ………………………………………………

7. Haben Sie eine der folgenden viralen Infektionen durchgemacht?

☐ FSME
☐ Ja ☐ Nein
Wenn ja, wann? ………………………………………………

☐ Gelbfieber
☐ Ja ☐ Nein
Wenn ja, wann? ………………………………………………

☐ Westnil-Virus
☐ Ja ☐ Nein
Wenn ja, wann? ………………………………………………

☐ Dengue
☐ Ja ☐ Nein
Wenn ja, wann? ………………………………………………

☐ Japanische Enzephalitis
☐ Ja ☐ Nein
Wenn ja, wann? ………………………………………………

8. Hatten Sie in den letzten 18 Monaten eine grippale Symptomatik mit Hautausschlägen und Bindehaut/Augen- Entzündung:

☐ Ja ☐ Nein
Wenn ja, wann? ………………………………………………

9. Ihre Anmerkungen

……………………………………………………………………………………..

…………………………………………………………………………………….

Vielen Dank für Ihre Mithilfe!
