# Supplementary material for: No molecular or serological evidence of Zikavirus infection among healthy blood donors living in or travelling to regions where Aedes albopictus circulates
Source: PLoS One. 2017 May 24;12(5):e0178175. doi: 10.1371/journal.pone.0178175 (PMC5443526; doi:10.1371/journal.pone.0178175)
Supplement: S2 File — (PDF) [file pone.0178175.s004.pdf]

Code: |__|__|__|__|

Case Report Form

Searching for evidence of Zika virus infection among blood donors in Tyrol, West Austria
(to be filled by the study participant)

Date of sample collection:

|__|__|-|__|__|-|__|__|__|__|

T T M M J J J J

1. Gender:
   ☐ Male ☐ Female

2. Age:
   ………… years

3. Have you been in any country in South America or the Caribbean in the last 18 months?

   ☐ Yes ☐ No
   If Yes, when? ……………………………………………………………….

4. Have you visited any country in East or Southeast Asia in the last 18 months?

   ☐ Yes ☐ No
   If yes, when? ……………………………………………………………….

5. Have you visited any of the following countries between April and October 2016: Italy, France, Spain, Greece, Croatia, Slovenia, Macedonia, Montenegro or Turkey?

   ☐ Yes ☐ No
   If yes, when? ………………………………………….
   If yes, where exactly (which city or island)? ……………………………………

6. Are you vaccinated against any of the following illnesses?
   ☐ Tick borne encephalitis

☐ Yes ☐ No
If yes, when? ………………………………………………

☐ Yellow fever
☐ Yes ☐ No
If yes, when? ………………………………………………

☐ Japanese encephalitis
☐ Yes ☐ No
If yes, when? ………………………………………………

7. Have you had any of the following infections in the past?
☐ Tick borne encephalitis
☐ Yes ☐ No
If yes, when? ………………………………………………

☐ Yellow fever
☐ Yes ☐ No
If yes, when? ………………………………………………

☐ West Nile fever
☐ Yes ☐ No
If yes, when? ………………………………………………

☐ Dengue fever
☐ Yes ☐ No
If yes, when? ………………………………………………

☐ Japanese encephalitis
☐ Yes ☐ No
If yes, when? ………………………………………………

8. Did you have any flu-like illness in the last 18 months associated with skin rash and conjunctivitis (red eye)?

☐ Yes ☐ No
If yes, when? ………………………………………………

9. Any remarks?
……………………………………………………………………………………………..

…………………………………………………………………………………………….

Thank you for participating!
