# Supplementary material for: No molecular or serological evidence of Zikavirus infection among healthy blood donors living in or travelling to regions where Aedes albopictus circulates
Source: PLoS One. 2017 May 24;12(5):e0178175. doi: 10.1371/journal.pone.0178175 (PMC5443526; doi:10.1371/journal.pone.0178175)
Supplement: S3 File — (PDF) [file pone.0178175.s005.pdf]

Landeck 19.9.16
2. Teil

Dade Behring Marburg GmbH
Postfach 11 49
D-35001 Marburg

IgG

28.10.16 12:03:45

Plattenreport: Rohwerte

Platten-ID:	0011	Riegel:	1 - 10
BES Version:	V3.3	Validierung:	-
Analyzer:	151549		
Testname:	Photometrieren	Einheit:	-
Cut Off:	-	Valid.bereich	
Cut Off Formel:	-		
Wiederh.bereich:	-		
Blankkorrektur	-		
Niveau Korrektur	-		
Wellenlängen M/R:	450 nm / 650 nm		
Testkit Nr.:	-		
Platte definiert am:	28.10.16 11:57		

Zika - Studie [signature]

!	Napfauffälligkeit		Ergebnis negativ
#	Keine Berechnung möglich	*	Ergebnis positiv
∞	Ergebnis zweifelhaft	?	Ergebnis in Wiederh.Ber.

IgG

	1	2	3	4	5	6	7	8	9	10	11	12
A	Kal. 0.423	96 0.113 0,267	304 0.142	78 0.058	19 0.024	296 0.021	279 0.013	017 0.025	115 0.080	132 0.262 0,619		
B	pos. 0.855 2,021	107 0.022 0,052	225 0.029	88 0.021	89 0.050	138 0.030	135 0.028	110 0.021	008 0.021	90 0.061		
C	neg. 0.030 0,064	191 0.027 0,063	303 0.016	89 0.041	49 0.019	285 0.085	290 0.023	37 0.030	018 0.036	249 0.017		
D	16 0.043 0,092	208 0.019 0,044	186 0.049	39 0.016	116 0.036	137 0.023	001 0.028	47 0.035	028 0.019	70 0.028		
E	26 0.021 0,049	250 0.022 0,052	190 0.021	69 0.023	286 0.022	139 0.021	119 0.027	102 0.047	038 0.054	60 0.035		
F	36 0.052 0,122	207 0.023 0,054	194 0.017	79 0.031	145 0.027	129 0.017	021 0.021	87 0.021	048 + 0.483 1,141	50 0.044		
G	46 0.029 0,068	254 0.015 0,035	192 0.040	103 0.014	298 0.023	176 0.025	031 0.028	97 0.017	058 0.044	90 0.041		
H	86 0.018 0,042	203 0.069 0,163	66 0.093	98 0.018	289 0.047	164 0.131	007 0.049	67 0.060	080 0.028	106 0.027		

Plate Flags:
Einzelfunktionsplatte

Ratio: < 0,8 neg.
≥ 0,8 - 1,1 GW
≥ 1,1 pos.

Analyzer Seriennummer: 151549

Londeck 19.9.16
2. Teil

Dade Behring Marburg GmbH
Postfach 11 49
D-35001 Marburg

IgM

28.10.16 12:08:56

Plattenreport: Rohwerte

Platten-ID:	0012	Riegel:	1 - 10
BES Version:	V3.3	Validierung:	-
Analyzer:	151549		
Testname:	Photometrieren	Einheit:	-
Cut Off:	-	Valid.bereich	
Cut Off Formel:	-		
Wiederh.bereich:	-		
Blankkorrektur	-		
Niveau Korrektur	-		
Wellenlängen M/R:	450 nm / 650 nm		
Testkit Nr.:	-		
Platte definiert am:	28.10.16 11:57		

Zika - Studie

[signature]

!	Napfauffälligkeit		Ergebnis negativ
#	Keine Berechnung möglich	*	Ergebnis positiv
∞	Ergebnis zweifelhaft	?	Ergebnis in Wiederh.Ber.

IgM	1	2	3	4	5	6	7	8	9	10	11	12
A	Cal?<br>0.467	96<br>0.025<br>0,053	304<br>0.027<br>0,057	78<br>0.077<br>0,164	19<br>0.085<br>0,182	296<br>0.049<br>0,104	279<br>0.056<br>0,119	017<br>0.033<br>0,070	115<br>0.048<br>0,102	132<br>0.029<br>0,062		
B	pos.<br>* 1.172<br>2,509	107<br>0.069<br>0,147	225<br>0.012<br>0,025	88<br>0.017<br>0,036	89<br>0.029<br>0,062	138<br>0.031<br>0,066	135<br>0.018<br>0,038	110<br>0.017<br>0,036	008<br>0.019<br>0,040	90<br>0.042<br>0,089		
C	neg.<br>0.030<br>0,064	191<br>0.013<br>0,027	303<br>0.025<br>0,053	89<br>0.019<br>0,040	49<br>0.086<br>0,184	285<br>0.030<br>0,064	290<br>0.015<br>0,032	37<br>0.030<br>0,064	018<br>0.043<br>0,092	249<br>0.038<br>0,081		
D	16<br>0.038<br>0,081	208<br>0.034<br>0,072	186<br>0.057<br>0,122	39<br>0.015<br>0,032	116<br>0.020<br>0,042	137<br>0.025<br>0,053	001<br>0.017<br>0,036	47<br>0.030<br>0,064	028<br>0.023<br>0,049	70<br>0.057<br>0,122		
E	26<br>0.305<br>0,653	250<br>0.035<br>0,074	190<br>0.015<br>0,032	69<br>0.047<br>0,100	286<br>0.015<br>0,032	139<br>0.015<br>0,032	119<br>0.021<br>0,044	102<br>0.013<br>0,027	038<br>0.047<br>0,100	60<br>0.026<br>0,055		
F	36<br>0.040<br>0,085	207<br>0.081<br>0,173	194<br>0.011<br>0,023	79<br>0.051<br>0,109	145<br>0.061<br>0,130	129<br>0.020<br>0,042	021<br>0.013<br>0,027	87<br>0.080<br>0,171	048<br>0.408<br>0,873	50<br>0.105<br>0,224		
G	46<br>0.027<br>0,057	254<br>0.042<br>0,089	192<br>0.039<br>0,083	103<br>0.012<br>0,025	298<br>0.124<br>0,265	176<br>0.013<br>0,027	031<br>0.014<br>0,029	97<br>0.032<br>0,068	058<br>0.020<br>0,042	40<br>0.025<br>0,053		
H	86<br>0.017<br>0,036	203<br>0.021<br>0,044	66<br>0.055<br>0,117	98<br>0.019<br>0,040	289<br>0.053<br>0,113	164<br>0.054<br>0,115	007<br>0.017<br>0,036	67<br>0.060<br>0,128	080<br>0.069<br>0,147	106<br>0.027<br>0,057		

Plate Flags:
Einzelfunktionsplatte

Ratio: < 0,8 neg.
≥ 0,8 - 1,1 GW
≥ 1,1 pos.

Analyzer Seriennummer: 151549

Brixlegg 14.4.2016

Dade Behring Marburg GmbH
Postfach 11 49
D-35001 Marburg

18.10.2016 13:36:17 Uhr

IgG

Plattenreport: Rohwerte

Platten-ID:	0012	Riegel:	1 - 10
BES Version:	V3.3	Validierung:	-
Analyzer:	202056		
Testname:	Photometrieren	Einheit:	-
Cut Off:	-	Valid.bereich	
Cut Off Formel:	-		
Wiederh.bereich:	-		
Blankkorrektur	-		
Niveau Korrektur	-		
Wellenlängen M/R:	450 nm / 650 nm		
Testkit Nr.:	-		
Platte definiert am:	18.10.2016 13:28 Uhr		

Zilke Elisa [signature]

!	Napfauffälligkeit		Ergebnis negativ
#	Keine Berechnung möglich	*	Ergebnis positiv
∞	Ergebnis zweifelhaft	?	Ergebnis in Wiederh.Ber.

IgG

	1	2	3	4	5	6	7	8	9	10	11	12
A	••••• Cal.<br>0.421	••••• 018<br>0.037<br>0,087	••••• 035<br>0.017<br>0,040	••••• 046<br>0.026<br>0,061	••••• 059<br>0.018<br>0,042	••••• 073<br>0.020<br>0,047	••••• 087<br>0.016<br>0,038	••••• 098<br>0.023<br>0,054	••••• 107<br>0.021<br>0,049	••••• 118<br>0.013<br>0,030		
B	••••• pos.<br>1.060<br>2,517	••••• 020<br>0.025<br>0,059	••••• 036<br>0.014<br>0,033	••••• 049<br>0.016<br>0,038	••••• 062<br>0.017<br>0,040	••••• 074<br>0.030<br>0,071	••••• 089<br>0.027<br>0,064	••••• 099<br>0.023<br>0,546	••••• 108<br>0.016<br>0,038	••••• 119<br>0.035<br>0,083		
C	••••• neg.<br>0.028<br>0,006	••••• 021<br>0.019<br>0,095	••••• 037<br>0.250<br>0,593	••••• 050<br>0.036<br>0,085	••••• 063<br>0.031<br>0,073	••••• 075<br>0.021<br>0,049	••••• 090<br>0.024<br>0,057	••••• 100<br>0.033<br>0,078	••••• 109<br>0.035<br>0,083	••••• 120<br>0.018<br>0,042		
D	••••• 006<br>0.013<br>0,030	••••• 025<br>0.046<br>0,109	••••• 038<br>0.146<br>0,346	••••• 051<br>0.036<br>0,085	••••• 064<br>0.058<br>0,137	••••• 077<br>0.014<br>0,033	••••• 091<br>0.026<br>0,061	••••• 101<br>0.022<br>0,052	••••• 112<br>0.023<br>0,054	••••• 121<br>0.056<br>0,133		
E	••••• 009<br>0.233<br>0,549	••••• 026<br>0.024<br>0,057	••••• 039<br>0.024<br>0,057	••••• 052<br>0.024<br>0,057	••••• 065<br>0.017<br>0,040	••••• 079<br>0.030<br>0,071	••••• 092<br>0.016<br>0,038	••••• 102<br>0.032<br>0,076	••••• 113<br>0.033<br>0,078	••••• 122<br>0.021<br>0,049		
F	••••• 011<br>0.020<br>0,047	••••• 028<br>0.039<br>0,092	••••• 042<br>0.020<br>0,047	••••• 053<br>0.065<br>0,154	••••• 067<br>0.024<br>0,057	••••• 081<br>0.022<br>0,052	••••• 094<br>0.037<br>0,087	••••• 103<br>0.055<br>0,130	••••• 114<br>0.031<br>0,073	••••• 130<br>0.024<br>0,057		
G	••••• 014<br>0.037<br>0,087	••••• 032<br>0.023<br>0,054	••••• 043<br>0.019<br>0,045	••••• 056<br>0.026<br>0,061	••••• 069<br>0.101<br>0,239	••••• 082<br>0.026<br>0,061	••••• 096<br>0.036<br>0,085	••••• 105<br>0.046<br>0,109	••••• 116<br>0.054<br>0,128	•••••<br>0.000		
H	••••• 016<br>0.067<br>0,159	••••• 033<br>0.016<br>0,038	••••• 045<br>0.026<br>0,061	••••• 058<br>0.044<br>0,104	••••• 072<br>0.018<br>0,042	••••• 086<br>0.032<br>0,076	••••• 097<br>0.032<br>0,076	••••• 106<br>0.030<br>0,071	••••• 117<br>0.023<br>0,054	•••••<br>-0.000		

Plate Flags:
Einzelfunktionsplatte

Ratio: < 0,8 neg.
≥ 0,8 - 1,1 grenzwertig
≥ 1,1 pos.

Analyzer Seriennummer: 202056

Brixlegg 14.9.2016

Dade Behring Marburg GmbH
Postfach 11 49
D-35001 Marburg

18.10.2016 13:34:32 Uhr

IgM

Plattenreport: Rohwerte

Platten-ID:	0011	Riegel:	1 - 10
BES Version:	V3.3	Validierung:	-
Analyzer:	202056		
Testname:	Photometrieren	Einheit:	-
Cut Off:	-	Valid.bereich	
Cut Off Formel:	-		
Wiederh.bereich:	-		
Blankkorrektur	-		
Niveau Korrektur	-		
Wellenlängen M/R:	450 nm / 650 nm		
Testkit Nr.:	-		
Platte definiert am:	18.10.2016 13:28 Uhr		

Zikke - Elisa

[signatures]

!	Napfauffälligkeit		Ergebnis negativ
#	Keine Berechnung möglich	*	Ergebnis positiv
∞	Ergebnis zweifelhaft	?	Ergebnis in Wiederh.Ber.

	IgM	2	3	4	5	6	7	8	9	10	11	12
A	Cal.<br>0.470	018<br>0.024<br>0,051	035<br>0.023<br>0,048	046<br>0.019<br>0,040	059<br>0.036<br>0,076	073<br>0.015<br>0,031	087<br>0.026<br>0,055	098<br>0.033<br>0,070	107<br>0.016<br>0,034	118<br>0.038<br>0,080		
B	pos.<br>1.005<br>1,138	020<br>0.023<br>0,048	036<br>0.019<br>0,040	049<br>0.014<br>0,029	062<br>0.021<br>0,044	074<br>0.020<br>0,042	089<br>0.023<br>0,048	099<br>0.143<br>0,304	108<br>0.020<br>0,042	119<br>0.022<br>0,046		
C	neg.<br>0.031<br>0,065	021<br>0.124<br>0,263	037<br>0.058<br>0,123	050<br>0.132<br>0,280	063<br>0.024<br>0,051	075<br>0.017<br>0,036	090<br>0.018<br>0,038	100<br>0.038<br>0,080	109<br>0.024<br>0,051	120<br>0.025<br>0,053		
D	006<br>0.027<br>0,057	025<br>0.048<br>0,102	038<br>0.018<br>0,038	051<br>0.020<br>0,042	064<br>0.017<br>0,036	077<br>0.022<br>0,046	091<br>0.018<br>0,038	101<br>0.042<br>[illegible]	112<br>0.039<br>0,082	121<br>0.020<br>0,042		
E	009<br>0.018<br>0,038	026<br>0.017<br>0,036	039<br>0.043<br>0,091	052<br>0.040<br>0,085	065<br>0.026<br>0,055	079<br>0.101<br>0,214	092<br>0.015<br>0,031	102<br>0.025<br>0,053	113<br>0.059<br>0,125	122<br>0.027<br>0,057		
F	011<br>0.011<br>0,023	028<br>0.032<br>[illegible]	042<br>0.017<br>0,036	053<br>0.022<br>0,046	067<br>0.048<br>0,102	081<br>0.031<br>0,065	094<br>0.017<br>0,036	103<br>0.039<br>0,082	114<br>0.026<br>0,055	130<br>0.255<br>0,542		
G	014<br>0.029<br>0,061	032<br>0.059<br>0,125	043<br>0.021<br>0,044	056<br>0.021<br>0,044	069<br>0.021<br>0,044	082<br>0.039<br>0,082	096<br>0.019<br>0,040	105<br>0.021<br>0,044	116<br>0.089<br>0,189	-0.001		
H	016<br>0.073<br>0,155	033<br>0.025<br>0,053	045<br>0.045<br>0,095	058<br>0.039<br>0,082	072<br>0.021<br>0,044	086<br>0.022<br>0,046	097<br>0.025<br>0,053	106<br>0.037<br>0,078	117<br>0.080<br>0,170	-0.000		

Plate Flags:

Einzelfunktionsplatte

Ratio: < 0,8 neg.
≥ 0,8-1,1 grenzwertig
≥ 1,1 pos.

Analyzer Seriennummer: 202056

Thiesse 29.8.2016

Dade Behring Marburg GmbH
Postfach 11 49
D-35001 Marburg

IgG

19.10.2016 12:21:45

Plattenreport: Rohwerte

Platten-ID:	0011	Riegel:	1 - 12
BES Version:	V3.3	Validierung:	-
Analyzer:	151549		
Testname:	Photometrieren	Einheit:	-
Cut Off:	-	Valid.bereich	
Cut Off Formel:	-		
Wiederh.bereich:	-		
Blankkorrektur	-		
Niveau Korrektur	-		
Wellenlängen M/R:	450 nm / 650 nm		
Testkit Nr.:	-		
Platte definiert am:	19.10.2016 11:58		

Zika - Elise

Gö. Ru.

!	Napfauffälligkeit		Ergebnis negativ
#	Keine Berechnung möglich	*	Ergebnis positiv
∞	Ergebnis zweifelhaft	?	Ergebnis in Wiederh.Ber.

	IgG 1	2	3	4	5	6	7	8	9	10	11	12
A	••••• Cal.<br>0.438	••••• 18<br>0.027<br>0,061	••••• 32<br>0.278<br>0,634	••••• 45<br>0.064<br>0,146	••••• 60<br>0.094<br>0,214	••••• 74<br>0.138<br>0,315	••••• 101<br>0.016<br>0,036	••••• 121<br>0.035<br>0,079	•••••<br>-0.000	•••••<br>-0.000	•••••<br>0.000	•••••<br>-0.000
B	••••• pos.<br>0.797<br>1,819	••••• 19<br>0.048<br>0,109	••••• 34<br>0.026<br>0,059	••••• 46<br>0.027<br>0,061	••••• 61<br>0.024<br>0,054	••••• 75<br>0.043<br>0,098	••••• 103<br>0.037<br>0,084	••••• 126<br>0.052<br>0,118	•••••<br>-0.000	•••••<br>-0.000	•••••<br>0.000	•••••<br>-0.000
C	••••• neg.<br>0.027<br>0,061	••••• 21<br>0.019<br>0,043	••••• 35<br>0.096<br>0,219	••••• 49<br>0.029<br>0,066	••••• 82<br>0.049<br>0,111	••••• 78<br>0.029<br>0,066	••••• 107<br>0.027<br>0,061	•••••<br>0.002	•••••<br>0.000	•••••<br>-0.000	•••••<br>0.000	•••••<br>-0.000
D	••••• 06<br>0.207<br>0,472	••••• 23<br>0.031<br>0,070	••••• 36<br>0.149<br>0,340	••••• 52<br>0.020<br>0,045	••••• 64<br>0.029<br>0,066	••••• 90<br>0.134<br>0,305	••••• 109<br>0.023<br>0,052	•••••<br>0.000	•••••<br>0.000	•••••<br>0.000	•••••<br>0.000	•••••<br>0.000
E	••••• 08<br>0.365<br>0,833	••••• 25<br>0.039<br>0,089	••••• 38<br>0.028<br>0,063	••••• 55<br>0.158<br>0,360	••••• 67<br>0.038<br>0,086	••••• 91<br>0.021<br>0,047	••••• 110<br>0.023<br>0,052	•••••<br>-0.000	•••••<br>0.000	•••••<br>-0.000	•••••<br>0.000	•••••<br>-0.000
F	••••• 10<br>0.024<br>0,054	••••• 26<br>0.100<br>0,228	••••• 41<br>0.038<br>0,086	••••• 56<br>0.146<br>0,333	••••• 68<br>0.081<br>0,184	••••• 94<br>0.103<br>0,235	••••• 117<br>0.015<br>0,034	•••••<br>0.000	•••••<br>0.000	•••••<br>0.000	•••••<br>0.000	•••••<br>0.000
G	••••• 12<br>0.019<br>0,043	••••• 28<br>0.054<br>0,123	••••• 42<br>0.078<br>0,178	••••• 58<br>0.020<br>0,045	••••• 69<br>0.052<br>0,118	••••• 95<br>0.104<br>0,237	••••• 119<br>0.053<br>0,121	•••••<br>0.000	•••••<br>0.000	•••••<br>0.000	•••••<br>0.000	•••••<br>-0.000
H	••••• 16<br>0.031<br>0,070	••••• 31<br>0.022<br>0,050	••••• 43<br>0.017<br>0,038	••••• 59<br>0.022<br>0,050	••••• 73<br>0.029<br>0,066	••••• 98<br>0.081<br>0,184	••••• 121<br>0.036<br>0,082	•••••<br>0.000	•••••<br>0.000	•••••<br>0.000	•••••<br>0.000	•••••<br>0.000

Plate Flags:
Einzelfunktionsplatte

Ratio: < 0,8 neg.
≥ 0,8 - 1,1 grenzwertig
≥ 1,1 pos.

Analyzer Seriennummer: 151549

Thiersee 29.8.2016

Dade Behring Marburg GmbH
Postfach 11 49
D-35001 Marburg

IgM

19.10.2016 12:09:36

Plattenreport: Rohwerte

Platten-ID:	0012	Riegel:	1 - 12
BES Version:	V3.3	Validierung:	-
Analyzer:	151549		
Testname:	Photometrieren	Einheit:	-
Cut Off:	-	Valid.bereich	
Cut Off Formel:	-		
Wiederh.bereich:	-		
Blankkorrektur	-		
Niveau Korrektur	-		
Wellenlängen M/R:	450 nm / 650 nm		
Testkit Nr.:	-		
Platte definiert am:	19.10.2016 11:58		

Zikka - Elisa

[signature]

!	Napfauffälligkeit	*	Ergebnis negativ
#	Keine Berechnung möglich	?	Ergebnis positiv
∞	Ergebnis zweifelhaft		Ergebnis in Wiederh.Ber.

	IgM	2	3	4	5	6	7	8	9	10	11	12
A	••••• Cal. 0.493	••••• 18 0.032 0,064	••••• 32 0.047 0,095	••••• 45 0.040 0,081	••••• 60 0.054 0,109	••••• 74 0.042 0,085	••••• 101 0.017 0,034	••••• 122 0.041 0,083	••••• -0.000	••••• -0.000	••••• -0.000	••••• -0.000
B	••••• pos. 1.199 2,452	••••• 19 0.020 0,040	••••• 34 0.040 0,081	••••• 46 0.016 0,032	••••• 61 0.085 0,172	••••• 45 0.019 0,038	••••• 103 0.035 0,070	••••• 126 0.013 0,026	••••• -0.000	••••• -0.000	••••• -0.000	••••• -0.000
C	••••• neg. 0.028 0,056	••••• 21 0.106 0,215	••••• 35 0.029 0,058	••••• 49 0.026 0,052	••••• 62 0.072 0,146	••••• 78 0.077 0,156	••••• 107 0.017 0,034	••••• 0.004	••••• -0.000	••••• 0.000	••••• -0.000	••••• -0.000
D	••••• 06 0.046 0,093	••••• 23 0.082 0,1[illegible]	••••• 36 0.020 0,040	••••• 52 0.025 0,050	••••• 64 0.017 0,034	••••• 90 0.165 0,334	••••• 109 0.018 0,036	••••• -0.000	••••• 0.000	••••• 0.000	••••• 0.000	••••• -0.000
E	••••• 08 0.283 0,574	••••• 25 0.030 0,060	••••• 38 0.139 0,281	••••• 55 0.027 0,054	••••• 67 0.025 0,050	••••• 91 0.033 0,0[illegible]	••••• 110 0.051 0,103	••••• -0.000	••••• -0.000	••••• -0.000	••••• -0.000	••••• -0.000
F	••••• 10 0.053 0,107	••••• 26 0.028 0,056	••••• 41 0.020 0,040	••••• 56 0.029 0,058	••••• 68 0.022 0,044	••••• 94 0.033 0,066	••••• 117 0.010 0,020	••••• -0.000	••••• -0.000	••••• -0.000	••••• -0.000	••••• -0.000
G	••••• 12 0.032 0,064	••••• 28 0.027 0,054	••••• 42 0.031 0,062	••••• 58 0.026 0,052	••••• 69 0.018 0,036	••••• 95 0.018 0,036	••••• 119 0.039 0,079	••••• -0.000	••••• -0.000	••••• -0.000	••••• -0.000	••••• -0.000
H	••••• 16 0.122 0,247	••••• 31 0.020 0,040	••••• 43 0.013 0,026	••••• 59 0.021 0,042	••••• 73 0.058 0,036	••••• 98 0.028 0,056	••••• 121 0.027 0,054	••••• -0.000	••••• -0.000	••••• -0.000	••••• -0.000	••••• -0.000

Plate Flags:
Einzelfunktionsplatte

Ratio: < 0,8 neg.
≥ 0,8-1,1 grenzwertig
≥ 1,1 pos.

Analyzer Seriennummer: 151549

Matrei 16.9.

Dade Behring Marburg GmbH
Postfach 11 49
D-35001 Marburg

IgM

20.10.2016 12:49:15 Uhr

Plattenreport: Rohwerte

Platten-ID:	0012	Riegel:	1 - 9
BES Version:	V3.3	Validierung:	-
Analyzer:	202056		
Testname:	Photometrieren	Einheit:	-
Cut Off:	-	Valid.bereich	
Cut Off Formel:	-		
Wiederh.bereich:	-		
Blankkorrektur	-		
Niveau Korrektur	-		
Wellenlängen M/R:	450 nm / 650 nm		
Testkit Nr.:	-		
Platte definiert am:	20.10.2016 12:38 Uhr		

Zikka - Elise

!	Napfauffälligkeit	*	Ergebnis negativ
#	Keine Berechnung möglich	?	Ergebnis positiv
∞	Ergebnis zweifelhaft		Ergebnis in Wiederh.Ber.

IgM

	1	2	3	4	5	6	7	8	9	10	11	12
A	Kal. 0.435	74 0.033 0,075	85 0.024 0,055	102 0.084 0,193	113 0.051 0,117	123 0.035 0,080	135 0.029 0,066	145 0.029 0,066	158 0.039 0,089			
B	pos. 1.151 ~~0,347~~	75 0.036 0,082	86 0.039 0,089	103 0.030 0,068	114 0.022 0,050	124 0.015 0,034	136 0.055 0,126	147 0.291 0,668	159 0.030 0,068			
C	neg. 0.024 0,055	76 0.015 0,034	89 0.020 0,045	104 0.019 0,043	115 0.012 0,027	125 0.087 0,2	138 0.015 0,034	148 0.019 0,043	[illegible] 0.050 0,114			
D	65 0.025 0,057	77 0.034 0,078	96 0.030 0,068	106 0.020 0,045	116 0.022 0,050	126 0.025 0,057	139 0.034 0,078	150 0.026 0,059	[illegible] 0.141 0,324			
E	66 0.018 0,041	80 0.163 0,374	98 0.034 0,078	107 0.029 0,066	117 0.015 0,034	127 0.033 0,095	140 0.021 0,048	153 0.044 0,101	152 0.030 0,068			
F	68 0.064 0,147	81 0.024 0,055	92 0.035 0,080	108 0.055 0,126	119 0.054 0,124	128 0.040 0,091	141 0.024 0,055	156 0.031 0,071	0.000			
G	70 0.017 0,039	82 0.030 0,068	99 0.037 0,085	109 0.013 0,029	120 0.017 0,039	131 0.021 0,048	143 0.016 0,036	154 0.028 0,064	0.000			
H	71 0.018 0,041	83 0.018 0,041	100 0.032 0,073	112 0.010 0,022	122 0.023 0,052	133 0.030 0,068	144 0.044 0,101	157 0.033 0,075	0.000			

2,645

Plate Flags:
Einzelfunktionsplatte

Ratio: < 0,8 neg.
≥ 0,8 - 1,1 GB
≥ 1,1 pos.

Malrei 16.9.

Dade Behring Marburg GmbH
Postfach 11 49
D-35001 Marburg

IgG

20.10.2016 12:49:03 Uhr

Plattenreport: Rohwerte

Platten-ID:	0011	Riegel:	1 - 9
BES Version:	V3.3	Validierung:	-
Analyzer:	202056		
Testname:	Photometrieren	Einheit:	-
Cut Off:	-	Valid.bereich	
Cut Off Formel:	-		
Wiederh.bereich:	-		
Blankkorrektur	-		
Niveau Korrektur	-		
Wellenlängen M/R:	450 nm / 650 nm		
Testkit Nr.:	-		
Platte definiert am:	20.10.2016 12:37 Uhr		

Zilka-Elise

!	Napfauffälligkeit		Ergebnis negativ
#	Keine Berechnung möglich	*	Ergebnis positiv
∞	Ergebnis zweifelhaft	?	Ergebnis in Wiederh.Ber.

	IgG	2	3	4	5	6	7	8	9	10	11	12
A	Col.<br>0.518	74<br>0.037<br>0,072	85<br>0.056<br>0,109	102<br>0.042<br>0,081	113<br>0.039<br>0,076	123<br>0.034<br>0,066	135<br>0.357<br>0,695	145<br>0.022<br>0,042	158<br>0.100<br>0,194			
B	pos.<br>1.205<br>2,348	75<br>0.080<br>0,155	86<br>0.017<br>0,033	103<br>0.018<br>0,035	114<br>0.023<br>0,044	124<br>0.077<br>0,150	136<br>0.089<br>0,173	147<br>0.020<br>0,038	159<br>0.030<br>0,058			
C	neg.<br>0.030<br>0,058	76<br>0.015<br>0,029	89<br>0.023<br>0,044	104<br>0.051<br>0,099	115<br>0.016<br>0,031	125<br>0.036<br>0,070	138<br>0.019<br>0,037	148<br>0.018<br>0,035	H16-25665K<br>0.052<br>0,101			
D	65<br>0.022<br>0,042	77<br>0.017<br>0,033	96<br>0.045<br>0,089	106<br>0.016<br>0,031	116<br>0.037<br>0,072	126<br>0.023<br>0,044	139<br>0.018<br>0,035	150<br>0.039<br>0,076	H16-25665V<br>0.127<br>0,247			
E	66<br>0.014<br>0,027	80<br>0.015<br>0,029	98<br>0.045<br>0,087	107<br>0.043<br>0,083	117<br>0.015<br>0,029	127<br>0.025<br>0,048	140<br>0.022<br>0,042	153<br>0.163<br>0,317	152<br>0.026<br>0,050			
F	68<br>0.020<br>0,038	81<br>0.024<br>0,046	92<br>0.045<br>0,087	108<br>0.034<br>0,066	119<br>0.018<br>0,035	128<br>0.042<br>0,081	141<br>0.023<br>0,044	156<br>0.025<br>0,048	0.000			
G	79<br>0.027<br>0,038	82<br>0.015<br>0,029	99<br>0.015<br>0,029	109<br>0.053<br>0,103	120<br>0.016<br>0,031	131<br>0.032<br>0,062	143<br>0.016<br>0,031	154<br>0.033<br>0,064	0.000			
H	71<br>0.028<br>0,054	83<br>0.076<br>0,148	100<br>0.025<br>0,048	112<br>0.023<br>0,044	122<br>0.017<br>0,033	133<br>0.028<br>0,054	144<br>0.017<br>0,033	157<br>0.033<br>0,064	0.000			

Plate Flags:

Einzelfunktionsplatte

Ratio: < 0,8 neg.
≥ 0,8–1,1 Grenzwertig
≥ 1,1 pos.

Wolters 12.10.16

Dade Behring Marburg GmbH
Postfach 11 49
D-35001 Marburg

IgM

21.10.2016 14:28:36 Uhr

Plattenreport: Rohwerte

Platten-ID:	0012	Riegel:	1 - 11
BES Version:	V3.3	Validierung:	-
Analyzer:	202056		
Testname:	Photometrieren	Einheit:	-
Cut Off:	-	Valid.bereich	
Cut Off Formel:	-		
Wiederh.bereich:	-		
Blankkorrektur	-		
Niveau Korrektur	-		
Wellenlängen M/R:	450 nm / 650 nm		
Testkit Nr.:	-		
Platte definiert am:	21.10.2016 14:18 Uhr		

Zika-Elisa

[illegible signature]

!	Napfauffälligkeit	*	Ergebnis negativ
#	Keine Berechnung möglich	?	Ergebnis positiv
∞	Ergebnis zweifelhaft		Ergebnis in Wiederh.Ber.

IgM

	1	2	3	4	5	6	7	8	9	10	11	12
A	Kal.<br>0.490	0013<br>0.779<br>1,589	0029<br>0.037<br>0,075	0046<br>0.042<br>0,085	0068<br>0.035<br>0,071	0080<br>0.051<br>0,104	0090<br>0.041<br>0,083	0107<br>0.050<br>0,102	0121<br>0.033<br>0,067	0135<br>0.028<br>0,057	0149<br>0.024<br>0,048	
B	pos.<br>1.174<br>2,395	0016<br>0.028<br>0,057	0031<br>0.042<br>0,085	0048<br>0.031<br>0,063	0070<br>0.035<br>0,071	0081<br>0.069<br>0,140	0091<br>0.060<br>0,122	0108<br>0.052<br>0,106	0123<br>0.038<br>0,077	0137<br>0.044<br>0,089	0151<br>0.041<br>0,083	
C	neg.<br>0.039<br>0,079	0018<br>0.031<br>0,063	0032<br>0.027<br>0,055	0049<br>0.059<br>0,120	0071<br>0.034<br>0,069	0083<br>0.036<br>0,073	0093<br>0.059<br>0,120	0109<br>0.033<br>0,067	0125<br>0.068<br>0,138	0139<br>0.034<br>0,069	0152<br>0.037<br>0,075	
D	0005<br>0.033<br>0,067	0021<br>0.038<br>0,077	0034<br>0.074<br>0,151	0052<br>0.029<br>0,059	0072<br>0.031<br>0,063	0085<br>0.038<br>0,077	0094<br>0.025<br>0,051	0110<br>0.029<br>0,059	0129<br>0.021<br>0,042	0142<br>0.101<br>0,206	0154<br>0.035<br>0,071	
E	0005<br>0.091<br>0,185	0022<br>0.030<br>0,061	0037<br>0.031<br>0,063	0055<br>0.032<br>0,065	0075<br>0.039<br>0,079	0086<br>0.038<br>0,077	0096<br>0.027<br>0,055	0113<br>0.043<br>0,087	0130<br>0.027<br>0,055	0143<br>0.037<br>0,075	0156<br>0.037<br>0,075	
F	0007<br>0.054<br>0,110	0025<br>0.039<br>0,079	0039<br>0.031<br>0,063	0059<br>0.029<br>0,059	0076<br>0.037<br>0,075	0087<br>0.032<br>0,065	0097<br>0.033<br>0,067	0115<br>0.045<br>0,091	0151<br>0.060<br>0,122	0144<br>0.058<br>0,118	0101<br>0.022<br>0,044	
G	0009<br>0.068<br>0,138	0026<br>0.028<br>0,057	0044<br>0.028<br>0,057	0061<br>0.055<br>0,112	0077<br>0.031<br>0,063	0088<br>0.146<br>0,297	0100<br>0.028<br>0,057	0116<br>0.021<br>0,042	0133<br>0.025<br>0,051	0145<br>0.141<br>0,287	0.000	
H	0010<br>0.040<br>0,081	0028<br>0.040<br>0,081	0045<br>0.029<br>0,059	0067<br>0.051<br>0,104	0079<br>0.032<br>0,065	0089<br>0.044<br>~~0,09~~ 0,089	0105<br>0.058<br>0,118	0119<br>0.027<br>0,055	0134<br>0.029<br>0,059	0148<br>0.025<br>0,051	0.000	

Plate Flags:
Einzelfunktionsplatte

Ratio: < 0,8 neg.
≥ 0,8 - 1,1 GW
≥ 1,1 pos.

Analyzer Seriennummer: 202056

Wattens 12.10.16

Dade Behring Marburg GmbH
Postfach 11 49
D-35001 Marburg

IgG

21.10.2016 14:25:03 Uhr

Plattenreport: Rohwerte

Platten-ID:	0011	Riegel:	1 - 11
BES Version:	V3.3	Validierung:	-
Analyzer:	202056		
Testname:	Photometrieren	Einheit:	-
Cut Off:	-	Valid.bereich	
Cut Off Formel:	-		
Wiederh.bereich:	-		
Blankkorrektur	-		
Niveau Korrektur	-		
Wellenlängen M/R:	450 nm / 650 nm		
Testkit Nr.:	-		
Platte definiert am:	21.10.2016 14:18 Uhr		

Elke-Elise [illegible]

!	Napfauffälligkeit	*	Ergebnis negativ
#	Keine Berechnung möglich		Ergebnis positiv
∞	Ergebnis zweifelhaft	?	Ergebnis in Wiederh.Ber.

IgG

	1	2	3	4	5	6	7	8	9	10	11	12
A	Cal.<br>0.510	0013<br>0.027<br>0,052	0029<br>0.096<br>0,188	0046<br>0.028<br>0,054	0068<br>0.098<br>0,192	0080<br>0.038<br>0,074	0090<br>0.039<br>0,076	0107<br>0.027<br>0,052	0121<br>0.060<br>0,117	0135<br>0.029<br>0,056	0149<br>0.034<br>0,066	
B	pos.<br>1.058<br>2,074	0016<br>0.022<br>0,043	0031<br>0.131<br>0,256	0048<br>0.040<br>0,078	0070<br>0.071<br>0,139	0081<br>0.034<br>0,066	0091<br>0.038<br>0,074	0108<br>0.052<br>0,101	0123<br>0.054<br>0,105	0157<br>0.044<br>0,086	0151<br>0.051<br>0,1	
C	neg.<br>0.038<br>0,074	0018<br>0.024<br>0,047	0032<br>0.029<br>0,056	0049<br>0.063<br>0,123	0071<br>0.020<br>0,039	0083<br>0.027<br>0,052	0093<br>0.033<br>0,064	0109<br>0.029<br>0,056	0125<br>0.052<br>0,101	0139<br>0.059<br>0,115	0152<br>0.038<br>0,074	
D	0003<br>0.035<br>0,068	0021<br>0.038<br>0,074	0054<br>0.023<br>0,045	0052<br>0.027<br>0,052	0072<br>0.042<br>0,082	0085<br>0.069<br>0,135	0094<br>0.058<br>0,113	0110<br>0.072<br>0,141	0129<br>0.021<br>0,041	0142<br>0.058<br>0,113	0154<br>0.032<br>0,062	
E	0005<br>0.025<br>0,049	0022<br>0.094<br>0,184	0037<br>0.095<br>0,186	0055<br>0.075<br>0,147	0075<br>0.067<br>0,131	0086<br>0.089<br>0,174	0096<br>0.034<br>0,066	0113<br>0.034<br>0,066	0130<br>0.082<br>0,160	0143<br>0.056<br>0,109	0156<br>0.051<br>0,1	
F	0007<br>0.023<br>0,045	0025<br>0.025<br>0,049	0039<br>0.031<br>0,060	0059<br>0.076<br>0,149	0076<br>0.033<br>0,064	0084<br>0.040<br>0,078	0097<br>0.044<br>0,086	0115<br>0.038<br>0,074	0151<br>0.037<br>0,042	0144<br>0.026<br>0,050	101<br>0.031<br>0,060	
G	0009<br>0.028<br>0,054	0026<br>0.034<br>0,066	0044<br>0.044<br>0,086	0061<br>0.070<br>0,137	0077<br>0.025<br>0,049	0088<br>0.044<br>0,086	0100<br>0.030<br>0,058	0116<br>0.050<br>0,098	0133<br>0.097<br>0,190	0145<br>0.048<br>0,094	0.000 (crossed out)	
H	0010<br>0.049<br>0,096	0028<br>0.032<br>0,062	0045<br>0.045<br>0,088	0067<br>0.024<br>0,047	0079<br>0.029<br>0,056	0089<br>0.109<br>0,213	0105<br>0.033<br>0,064	0119<br>0.035<br>0,068	0134<br>0.029<br>0,056	0148<br>0.043<br>0,084	0.000 (crossed out)	

Plate Flags:
Einzelfunktionsplatte

Ratio: <0,8 neg.
≥ 0,8 - 1,1 GW
≥ 1,1 pos.

Analyzer Seriennummer: 202056

Lenz 27.9.16

Dade Behring Marburg GmbH
Postfach 11 49
D-35001 Marburg

IgG

25.10.16 12:25:46

Plattenreport: Rohwerte

Platten-ID:	0011	Riegel:	1 - 10
BES Version:	V3.3	Validierung:	-
Analyzer:	151549		
Testname:	Photometrieren	Einheit:	-
Cut Off:	-	Valid.bereich	
Cut Off Formel:	-		
Wiederh.bereich:	-		
Blankkorrektur	-		
Niveau Korrektur	-		
Wellenlängen M/R:	450 nm / 650 nm		
Testkit Nr.:	-		
Platte definiert am:	25.10.16 12:10		

Zika-Studie

[signature]

!	Napfauffälligkeit	*	Ergebnis negativ
#	Keine Berechnung möglich	?	Ergebnis positiv
∞	Ergebnis zweifelhaft		Ergebnis in Wiederh.Ber.

IgG

	1	2	3	4	5	6	7	8	9	10	11	12
A	Cal.<br>0.388	127<br>0.014<br>0,036	137<br>0.023<br>0,059	149<br>0.031<br>0,079	165<br>0.025<br>0,064	162<br>0.051<br>0,131	168<br>0.515<br>1,327	194<br>0.020<br>0,051	199<br>0.031<br>0,079	206<br>0.033<br>0,085		
B	pos.<br>0.677<br>1,744	123<br>0.020<br>0,051	136<br>0.012<br>0,030	153<br>0.018<br>0,046	164<br>0.016<br>0,041	185<br>0.029<br>0,074	171<br>0.041<br>0,105	191<br>0.028<br>0,072	195<br>0.044<br>0,113	196<br>0.030<br>0,077		
C	neg.<br>0.024<br>0,061	122<br>0.014<br>0,036	133<br>0.023<br>0,059	152<br>0.032<br>0,082	167<br>0.019<br>0,048	181<br>0.023<br>0,059	190<br>0.060<br>0,154	184<br>0.018<br>0,046	200<br>0.013<br>0,033	13/Wellens/WH<br>0.021		
D	131<br>0.059<br>0,152	151<br>0.013<br>0,033	135<br>0.023<br>0,059	148<br>0.019<br>0,048	172<br>0.019<br>0,048	178<br>0.016<br>0,041	189<br>0.018<br>0,046	180<br>0.037<br>0,095	193<br>0.022<br>0,056	0.000		
E	128<br>0.016<br>0,041	144<br>0.016<br>0,041	134<br>0.303<br>0,780	147<br>0.022<br>0,056	169<br>0.020<br>0,051	177<br>0.076<br>0,195	197<br>0.017<br>0,043	209<br>0.022<br>0,056	202<br>0.032<br>0,082	-0.000		
F	126<br>0.019<br>0,048	140<br>0.017<br>0,043	158<br>0.078<br>0,201	146<br>0.012<br>0,030	156<br>0.028<br>0,072	176<br>0.019<br>0,048	201<br>0.024<br>0,061	204<br>0.012<br>0,030	210<br>0.309<br>0,796	-0.000		
G	130<br>0.021<br>0,054	143<br>0.166<br>0,427	157<br>0.014<br>0,036	141<br>0.027<br>0,069	160<br>0.104<br>0,268	175<br>0.046<br>0,118	186<br>0.021<br>0,054	203<br>0.019<br>0,048	207<br>0.019<br>0,048	0.000		
H	124<br>0.011<br>0,028	138<br>0.036<br>0,092	154<br>0.081<br>0,208	170<br>0.017<br>0,043	159<br>0.056<br>0,144	174<br>0.020<br>0,051	188<br>0.013<br>0,033	198<br>0.138<br>0,355	208<br>0.023<br>0,059	-0.000		

Plate Flags:
Einzelfunktionsplatte

Ratio: < 0,8 neg.
≥ 0,8 - 1,1 GW
≥ 1,1 pos.

Analyzer Seriennummer: 151549

[illegible] 27.9.16

Dade Behring Marburg GmbH
Postfach 11 49
D-35001 Marburg

IgM

25.10.16 12:16:39

Plattenreport: Rohwerte

Platten-ID:	0012	Riegel:	1 - 10
BES Version:	V3.3	Validierung:	-
Analyzer:	151549		
Testname:	Photometrieren	Einheit:	-
Cut Off:	-	Valid.bereich	
Cut Off Formel:	-		
Wiederh.bereich:	-		
Blankkorrektur	-		
Niveau Korrektur	-		
Wellenlängen M/R:	450 nm / 650 nm		
Testkit Nr.:	-		
Platte definiert am:	25.10.16 12:10		

Zika - Studie

!	Napfauffälligkeit		Ergebnis negativ
#	Keine Berechnung möglich	*	Ergebnis positiv
∞	Ergebnis zweifelhaft	?	Ergebnis in Wiederh.Ber.

IgM

	1	2	3	4	5	6	7	8	9	10	11	12
A	Col.<br>0.302	127<br>0.014<br>0,046	137<br>0.013<br>0,043	149<br>0.013<br>0,043	165<br>0.101<br>0,334	162<br>0.021<br>0,069	168<br>0.022<br>0,072	194<br>0.028<br>0,092	199<br>0.019<br>0,062	206<br>0.015<br>0,049		
B	pos.<br>0.879<br>2,910	123<br>0.016<br>0,052	136<br>0.019<br>0,062	153<br>0.009<br>0,029	164<br>0.010<br>0,033	185<br>0.014<br>0,046	171<br>0.014<br>0,046	191<br>0.041<br>0,135	195<br>0.013<br>0,043	196<br>0.053<br>0,175		
C	neg.<br>0.017<br>0,056	122<br>0.028<br>0,092	133<br>0.020<br>0,066	152<br>0.018<br>0,059	167<br>0.012<br>0,039	181<br>0.010<br>0,033	190<br>0.022<br>0,072	184<br>0.011<br>0,036	200<br>0.031<br>0,102	13/Wettens/WH<br>0.520<br>1,721		
D	131<br>0.016<br>0,052	151<br>0.024<br>0,079	155<br>0.013<br>0,043	148<br>0.008<br>0,026	172<br>0.010<br>0,033	178<br>0.017<br>0,056	189<br>0.013<br>0,043	180<br>0.026<br>0,086	193<br>0.019<br>0,062	0.000		
E	128<br>0.012<br>0,039	144<br>0.023<br>0,076	134<br>0.051<br>0,168	147<br>0.018<br>0,059	169<br>0.009<br>0,029	177<br>0.020<br>0,066	197<br>0.025<br>0,082	209<br>0.019<br>0,062	202<br>0.014<br>0,046	-0.000		
F	126<br>0.013<br>0,043	140<br>0.032<br>0,105	158<br>0.011<br>0,034	146<br>0.012<br>0,039	156<br>0.073<br>0,241	176<br>0.025<br>0,082	201<br>0.019<br>0,062	204<br>0.016<br>0,052	210<br>0.026<br>0,086	-0.000		
G	150<br>0.026<br>0,086	143<br>0.010<br>0,033	157<br>0.045<br>0,149	141<br>0.115<br>0,380	160<br>0.014<br>0,046	175<br>1.423<br>4,711	186<br>0.029<br>0,096	203<br>0.019<br>0,062	207<br>0.012<br>0,039	0.000		
H	124<br>0.016<br>0,052	138<br>0.017<br>0,056	154<br>0.028<br>0,092	170<br>0.015<br>0,049	159<br>0.029<br>0,096	174<br>0.025<br>0,082	188<br>0.017<br>0,056	198<br>0.012<br>0,039	208<br>0.045<br>0,149	-0.000		

Plate Flags:

Einzelfunktionsplatte

Ratio: < 0,8 neg.
≥ 0,8 - 1,1 GW
≥ 1,1 pos.

Analyzer Seriennummer: 151549

Lienz 27.9.16 (1. Teil)

Dade Behring Marburg GmbH
Postfach 11 49
D-35001 Marburg

IgG

24.10.2016 12:33:57 Uhr

Plattenreport: Rohwerte

Platten-ID:	0011	Riegel:	1 - 11
BES Version:	V3.3	Validierung:	-
Analyzer:	202056		
Testname:	Photometrieren	Einheit:	-
Cut Off:	-	Valid.bereich	
Cut Off Formel:	-		
Wiederh.bereich:	-		
Blankkorrektur	-		
Niveau Korrektur	-		
Wellenlängen M/R:	450 nm / 650 nm		
Testkit Nr.:	-		
Platte definiert am:	24.10.2016 12:24 Uhr		

Zika – Studie

[signature]

!	Napfauffälligkeit		Ergebnis negativ
#	Keine Berechnung möglich	*	Ergebnis positiv
∞	Ergebnis zweifelhaft	?	Ergebnis in Wiederh.Ber.

	IgG 1	2	3	4	5	6	7	8	9	10	11	12
A	Cal.<br>0.456	14<br>0.029<br>0,063	24<br>0.078<br>0,171	47<br>0.018<br>0,039	32<br>0.022<br>0,048	52<br>0.017<br>0,037	75<br>0.026<br>0,131	92<br>0.041<br>0,089	109<br>0.057<br>0,125	118<br>0.070<br>0,153	112<br>0.024<br>0,052	
B	pos.<br>0.886<br>1,942	13<br>0.018<br>0,039	26<br>0.019<br>0,041	38<br>0.013<br>0,028	72<br>0.045<br>0,098	51<br>0.019<br>0,041	69<br>0.044<br>0,096	90<br>0.026<br>0,057	107<br>0.021<br>0,021	102<br>0.018<br>0,039	114<br>0.023<br>0,050	
C	neg.<br>0.026<br>0,057	08<br>0.019<br>0,041	29<br>0.020<br>0,043	37<br>0.028<br>0,061	67<br>0.113<br>0,247	48<br>0.017<br>0,037	73<br>0.023<br>0,050	89<br>0.020<br>0,043	105<br>0.016<br>0,035	103<br>0.047<br>0,103	129<br>0.017<br>0,037	
D	22<br>0.012<br>0,026	16<br>0.110<br>0,241	27<br>0.026<br>0,057	36<br>0.028<br>0,061	58<br>0.012<br>0,026	87<br>0.034<br>0,074	71<br>0.048<br>0,105	85<br>0.034<br>0,074	95<br>0.012<br>0,026	116<br>0.039<br>0,085	0.005	
E	20<br>0.015<br>0,032	12<br>0.020<br>0,043	21<br>0.014<br>0,030	39<br>0.017<br>0,037	64<br>0.083<br>0,182	84<br>0.019<br>0,041	70<br>0.014<br>0,030	88<br>0.073<br>0,160	100<br>0.028<br>0,061	117<br>0.017<br>0,037	-0.000	
F	17<br>0.246<br>0,539	33<br>0.096<br>0,210	25<br>0.080<br>0,175	41<br>0.017<br>0,037	60<br>0.022<br>0,048	83<br>0.024<br>0,052	110<br>0.016<br>0,035	86<br>0.031<br>0,067	91<br>0.033<br>0,072	113<br>0.023<br>0,050	0.000	
G	18<br>0.035<br>0,076	31<br>0.019<br>0,041	19<br>0.023<br>0,050	34<br>0.011<br>0,024	55<br>0.018<br>0,039	76<br>0.127<br>0,278	93<br>0.037<br>0,081	111<br>0.034<br>0,074	120<br>0.021<br>0,046	106<br>0.018<br>0,039	0.000	
H	15<br>0.025<br>0,054	28<br>0.026<br>0,057	50<br>0.019<br>0,041	35<br>0.019<br>0,041	54<br>0.015<br>0,032	74<br>0.021<br>0,046	96<br>0.083<br>0,182	108<br>0.015<br>0,032	125<br>0.028<br>0,061	115<br>0.035<br>0,076	0.000	

Plate Flags:

Einzelfunktionsplatte

Ratio: < 0,8 neg.
≥ 0,8 – 1,1 GW
≥ 1,1 pos.

Kurz 27.9.16 (1. Teil)

Dade Behring Marburg GmbH
Postfach 11 49
D-35001 Marburg

IgM

24.10.2016 12:39:52 Uhr

Plattenreport: Rohwerte

Platten-ID:	0012	Riegel:	1 - 11
BES Version:	V3.3	Validierung:	-
Analyzer:	202056		
Testname:	Photometrieren	Einheit:	-
Cut Off:	-	Valid.bereich	
Cut Off Formel:	-		
Wiederh.bereich:	-	Zika – Studie	
Blankkorrektur	-		
Niveau Korrektur	-		
Wellenlängen M/R:	450 nm / 650 nm		
Testkit Nr.:	-		
Platte definiert am:	24.10.2016 12:25 Uhr		

!	Napfauffälligkeit		Ergebnis negativ
#	Keine Berechnung möglich	*	Ergebnis positiv
∞	Ergebnis zweifelhaft	?	Ergebnis in Wiederh.Ber.

IgM

	1	2	3	4	5	6	7	8	9	10	11	12
A	Col.<br>0.514	14<br>0.022<br>0,042	24<br>0.098<br>0,190	47<br>0.018<br>0,035	32<br>0.062<br>0,120	52<br>0.019<br>0,036	75<br>0.035<br>0,068	92<br>0.017<br>0,033	101<br>0.045<br>0,087	118<br>0.018<br>0,035	112<br>0.044<br>0,085	
B	Pos.<br>1.274<br>2,478	13<br>0.086<br>0,167	26<br>0.089<br>0,173	38<br>0.026<br>0,050	72<br>0.031<br>0,060	51<br>0.029<br>0,056	69<br>0.019<br>0,036	90<br>0.024<br>0,046	107<br>0.059<br>0,114	102<br>0.027<br>0,052	114<br>0.027<br>0,052	
C	neg.<br>0.034<br>0,066	08<br>0.027<br>0,052	29<br>0.037<br>0,071	37<br>0.033<br>0,064	67<br>0.176<br>0,342	48<br>0.023<br>0,044	73<br>0.044<br>0,085	89<br>0.025<br>0,048	105<br>0.023<br>0,044	103<br>0.025<br>0,048	129<br>0.012<br>0,023	
D	22<br>0.031<br>0,060	16<br>0.060<br>0,116	27<br>0.033<br>0,064	36<br>0.036<br>0,070	58<br>0.019<br>0,036	87<br>0.021<br>0,040	71<br>0.078<br>0,151	85<br>0.049<br>0,095	95<br>0.021<br>0,040	116<br>0.015<br>0,029	0.008	
E	20<br>0.025<br>0,048	12<br>0.026<br>0,050	21<br>0.015<br>0,029	39<br>0.031<br>0,060	64<br>0.024<br>0,046	84<br>0.016<br>0,031	70<br>0.094<br>0,182	88<br>0.054<br>0,105	100<br>0.023<br>0,044	117<br>0.013<br>0,025	0.001	
F	17<br>0.104<br>0,201	33<br>0.014<br>0,027	25<br>0.036<br>0,070	41<br>0.019<br>0,036	62<br>0.115<br>0,223	83<br>0.020<br>0,038	110<br>0.029<br>0,056	86<br>0.022<br>0,042	91<br>0.026<br>0,050	113<br>0.021<br>0,040	0.000	
G	18<br>0.026<br>0,050	31<br>0.017<br>0,033	19<br>0.016<br>0,031	34<br>0.020<br>0,038	55<br>0.042<br>0,081	76<br>0.025<br>0,048	93<br>0.013<br>0,025	111<br>0.021<br>0,040	120<br>0.029<br>0,056	106<br>0.014<br>0,027	0.000	
H	15<br>0.043<br>0,083	28<br>0.014<br>0,027	50<br>0.014<br>0,027	35<br>0.017<br>0,033	54<br>0.016<br>0,031	74<br>0.021<br>0,040	96<br>0.024<br>0,046	108<br>0.026<br>0,050	125<br>0.028<br>0,054	115<br>0.016<br>0,031	0.000	

Plate Flags:
Einzelfunktionsplatte

Ratio: < 0,8 neg.
≥ 0,8 – 1,1 GW
≥ 1,1 pos.

Analyzer Seriennummer: 202056

Niederndorf / Kufstein
30.10.16

Dade Behring Marburg GmbH
Postfach 11 49
D-35001 Marburg

IgM

07.11.2016 12:49:56 Uhr

Plattenreport: Rohwerte

Platten-ID:	0012	Riegel:	1 - 12
BES Version:	V3.3	Validierung:	-
Analyzer:	202056		
Testname:	Photometrieren	Einheit:	-
Cut Off:	-	Valid.bereich	
Cut Off Formel:	-		
Wiederh.bereich:	-		
Blankkorrektur	-		
Niveau Korrektur	-		
Wellenlängen M/R:	450 nm / 650 nm		
Testkit Nr.:	-		
Platte definiert am:	07.11.2016 12:38 Uhr		

Zilke-Stecker [illegible]

!	Napfauffälligkeit	*	Ergebnis negativ
#	Keine Berechnung möglich		Ergebnis positiv
∞	Ergebnis zweifelhaft	?	Ergebnis in Wiederh.Ber.

IgM

	1	2	3	4	5	6	7	8	9	10	11	12
A	Pol.<br>0.380	118<br>0.013<br>0,034	99<br>0.020<br>0,052	15<br>0.035<br>0,092	96<br>0.021<br>0,055	03<br>0.023<br>0,060	71<br>0.035<br>0,092	55<br>0.036<br>0,094	48<br>0.013<br>0,034	39<br>0.020<br>0,052	136<br>0.035<br>0,092	156<br>0.027<br>0,071
B	pos.<br>0.946<br>2,489	114<br>0.017<br>0,044	100<br>0.026<br>0,068	24<br>0.027<br>0,071	79<br>0.022<br>0,057	20<br>0.035<br>0,092	64<br>0.042<br>0,110	40<br>0.032<br>0,084	49<br>0.020<br>0,052	27<br>0.021<br>0,055	133<br>0.018<br>0,047	159<br>0.096<br>0,252
C	neg.<br>0.025<br>0,065	111<br>0.019<br>0,05	103<br>0.019<br>0,05	26<br>0.042<br>0,110	78<br>0.045<br>0,118	30<br>0.020<br>0,052	75<br>0.022<br>0,057	56<br>0.016<br>0,042	43<br>0.018<br>0,047	31<br>0.018<br>0,047	162<br>0.016<br>0,042	155<br>0.019<br>0,05
D	130<br>0.035<br>0,092	96<br>0.017<br>0,044	106<br>0.016<br>0,042	112<br>0.018<br>0,047	23<br>0.014<br>0,036	68<br>0.018<br>0,047	58<br>0.036<br>0,094	57<br>0.024<br>0,063	47<br>0.028<br>0,073	151<br>0.019<br>0,05	163<br>0.080<br>0,210	164<br>0.030<br>0,078
E	113<br>0.164<br>0,431	50<br>0.036<br>0,094	131<br>0.020<br>0,052	107<br>0.041<br>0,107	24<br>0.016<br>0,042	63<br>0.025<br>0,065	76<br>0.019<br>0,05	51<br>0.023<br>0,060	42<br>0.057<br>0,15	134<br>0.020<br>0,052	139<br>0.016<br>0,042	141<br>0.043<br>0,113
F	115<br>0.011<br>0,028	97<br>0.015<br>0,039	127<br>0.031<br>0,081	83<br>0.081<br>0,213	17<br>0.021<br>0,055	65<br>0.025<br>0,065	53<br>0.030<br>0,078	52<br>0.019<br>0,05	41<br>0.025<br>0,065	132<br>0.046<br>0,121	157<br>0.015<br>0,039	165<br>0.161<br>0,423
G	120<br>0.011<br>0,028	110<br>0.017<br>0,044	123<br>0.014<br>0,036	89<br>0.017<br>0,044	21<br>0.028<br>0,073	67<br>0.030<br>0,078	06<br>0.024<br>0,063	66<br>0.016<br>0,042	32<br>0.025<br>0,065	135<br>0.021<br>0,055	154<br>0.022<br>0,057	0.000
H	124<br>0.079<br>0,207	109<br>0.015<br>0,039	04<br>0.017<br>0,044	08<br>0.035<br>0,092	18<br>0.014<br>0,036	60<br>0.035<br>0,092	59<br>0.072<br>0,189	45<br>0.035<br>0,092	34<br>0.013<br>0,034	137<br>0.030<br>0,078	138<br>0.037<br>0,097	0.000

Plate Flags:
Einzelfunktionsplatte

Ratio: < 0,8 neg.
≥ 0,8 - 1,1 GW
≥ 1,1 pos.

Niederndorf / Kufstein
30.10.16

Dade Behring Marburg GmbH
Postfach 11 49
D-35001 Marburg

IgG

07.11.2016 12:43:11 Uhr

Plattenreport: Rohwerte

Platten-ID:	0011	Riegel:	1 - 12
BES Version:	V3.3	Validierung:	-
Analyzer:	202056		
Testname:	Photometrieren	Einheit:	-
Cut Off:	-	Valid.bereich	
Cut Off Formel:	-		
Wiederh.bereich:	-		
Blankkorrektur	-		
Niveau Korrektur	-		
Wellenlängen M/R:	450 nm / 650 nm		
Testkit Nr.:	-		
Platte definiert am:	07.11.2016 12:38 Uhr		

Zilke - Studie

!	Napfauffälligkeit		Ergebnis negativ
#	Keine Berechnung möglich	*	Ergebnis positiv
∞	Ergebnis zweifelhaft	?	Ergebnis in Wiederh.Ber.

IgG

	1	2	3	4	5	6	7	8	9	10	11	12
A	Kal-<br>0.426	118<br>0.044<br>0,103	99<br>0.039<br>0,091	15<br>0.033<br>0,077	96<br>0.040<br>0,093	03<br>0.049<br>0,115	71<br>0.027<br>0,063	55<br>0.021<br>0,049	48<br>0.020<br>0,046	39<br>0.019<br>0,044	136<br>0.015<br>0,035	156<br>0.026<br>0,061
B	pos-<br>0.839<br>1,969	114<br>0.029<br>0,068	100<br>0.115<br>0,269	24<br>0.016<br>0,037	79<br>0.035<br>0,082	20<br>0.052<br>0,122	64<br>0.047<br>0,110	40<br>0.022<br>0,051	49<br>0.016<br>0,037	27<br>0.032<br>0,075	133<br>0.014<br>0,032	159<br>0.054<br>0,126
C	neg.-<br>0.028<br>0,065	111<br>0.040<br>0,093	103<br>0.042<br>0,098	26<br>0.037<br>0,086	78<br>0.027<br>0,063	30<br>0.024<br>0,056	75<br>0.021<br>0,049	56<br>0.108<br>0,253	43<br>0.087<br>0,204	31<br>0.017<br>0,039	162<br>0.017<br>0,039	155<br>0.025<br>0,058
D	130<br>0.029<br>0,068	96<br>0.023<br>0,053	106<br>0.020<br>0,046	112<br>0.078<br>0,183	23<br>0.020<br>0,046	68<br>0.031<br>0,072	58<br>0.014<br>0,032	57<br>0.022<br>0,051	47<br>0.020<br>0,046	151<br>0.014<br>0,032	163<br>0.061<br>0,143	164<br>0.016<br>0,037
E	113<br>0.031<br>0,072	50<br>0.034<br>0,078	131<br>0.023<br>0,053	107<br>0.069<br>0,161	24<br>0.029<br>0,068	63<br>0.023<br>0,053	76<br>0.030<br>0,070	51<br>0.028<br>0,065	42<br>0.095<br>0,223	134<br>0.022<br>0,051	139<br>0.028<br>0,065	141<br>0.030<br>0,070
F	115<br>0.020<br>0,046	97<br>0.013<br>0,030	127<br>0.043<br>0,100	83<br>0.059<br>0,138	17<br>0.012<br>0,028	65<br>0.147<br>0,345	53<br>0.040<br>0,093	52<br>0.015<br>0,035	41<br>0.033<br>0,077	132<br>0.020<br>0,046	157<br>0.018<br>0,042	165<br>0.024<br>0,056
G	120<br>0.021<br>0,049	110<br>0.017<br>0,039	123<br>0.017<br>0,039	89<br>0.082<br>0,192	21<br>0.033<br>0,077	67<br>0.039<br>0,091	06<br>0.020<br>0,046	66<br>0.032<br>0,075	32<br>0.034<br>0,079	135<br>0.029<br>0,068	154<br>0.032<br>0,075	0.000
H	124<br>0.016<br>0,037	109<br>0.034<br>0,079	04<br>0.026<br>0,061	08<br>0.031<br>0,072	18<br>0.022<br>0,051	60<br>0.029<br>0,068	54<br>0.035<br>0,082	45<br>0.055<br>0,129	34<br>0.024<br>0,056	137<br>0.048<br>0,112	158<br>0.017<br>0,039	0.000

Plate Flags:
Einzelfunktionsplatte

Ratio: < 0,8 neg.
≥ 0,8-1,1 GW
≥ 1,1 pos.

Analyzer Seriennummer: 202056

Landeck 19.9.16

Dade Behring Marburg GmbH
Postfach 11 49
D-35001 Marburg

IgM

27.10.2016 12:48:03 Uhr

Plattenreport: Rohwerte

Platten-ID:	0012	Riegel:	1 - 11
BES Version:	V3.3	Validierung:	-
Analyzer:	202056		
Testname:	Photometrieren	Einheit:	-
Cut Off:	-	Valid.bereich	
Cut Off Formel:	-		
Wiederh.bereich:	-		
Blankkorrektur	-		
Niveau Korrektur	-		
Wellenlängen M/R:	450 nm / 650 nm		
Testkit Nr.:	-		
Platte definiert am:	27.10.2016 12:36 Uhr		

Zika-Studie [signature]

!	Napfauffälligkeit	*	Ergebnis negativ
#	Keine Berechnung möglich		Ergebnis positiv
∞	Ergebnis zweifelhaft	?	Ergebnis in Wiederh.Ber.

IgM

	1	2	3	4	5	6	7	8	9	10	11	12
A	•••••<br>Cal.<br>0.477	•••••<br>200<br>0.035	•••••<br>189<br>0.021	•••••<br>205<br>0.033	•••••<br>147<br>0.046	•••••<br>291<br>0.028	•••••<br>83<br>0.051	•••••<br>101<br>0.022	•••••<br>45<br>0.013	•••••<br>33<br>0.013	•••••<br>62<br>0.033	
B	•••••<br>pos.<br>1.123	•••••<br>153<br>0.038	•••••<br>264<br>0.145	•••••<br>219<br>0.028	•••••<br>302<br>0.030	•••••<br>166<br>0.027	•••••<br>73<br>0.031	•••••<br>64<br>0.018	•••••<br>35<br>0.093	•••••<br>43<br>0.014	•••••<br>42<br>0.057	
C	•••••<br>neg.<br>0.035	•••••<br>154<br>0.050	•••••<br>294<br>0.028	•••••<br>167<br>0.022	•••••<br>206<br>0.146	•••••<br>270<br>0.012	•••••<br>111<br>0.022	•••••<br>44<br>0.019	•••••<br>25<br>0.024	•••••<br>63<br>0.012	•••••<br>22<br>0.022	
D	•••••<br>172<br>0.026	•••••<br>175<br>0.020	•••••<br>224<br>0.035	•••••<br>160<br>0.020	•••••<br>278<br>0.031	•••••<br>152<br>0.027	•••••<br>108<br>0.036	•••••<br>15<br>0.018	•••••<br>06<br>0.019	•••••<br>72<br>0.014	•••••<br>168<br>0.027	WH
E	•••••<br>162<br>0.030	•••••<br>127<br>0.023	•••••<br>237<br>0.036	•••••<br>150<br>0.016	•••••<br>281<br>0.031	•••••<br>174<br>0.017	•••••<br>128<br>0.033	•••••<br>05<br>0.052	•••••<br>95<br>0.018	•••••<br>92<br>0.018	•••••<br>175<br>+1.749<br>2,666	WH
F	•••••<br>221<br>0.031	•••••<br>161<br>0.029	•••••<br>182<br>0.426<br>0,893	•••••<br>148<br>0.060	•••••<br>274<br>0.018	•••••<br>142<br>0.020	•••••<br>14<br>0.053	•••••<br>94<br>0.051	•••••<br>85<br>0.115	•••••<br>03<br>0.022	•••••<br>0.005	
G	•••••<br>222<br>0.028	•••••<br>196<br>0.024	•••••<br>141<br>0.039	•••••<br>168<br>0.153	•••••<br>234<br>0.017	•••••<br>151<br>0.221	•••••<br>130<br>0.067	•••••<br>84<br>0.043	•••••<br>75<br>0.018	•••••<br>13<br>0.051	•••••<br>0.000	
H	•••••<br>184<br>0.024	•••••<br>183<br>0.020	•••••<br>201<br>0.021	•••••<br>155<br>0.043	•••••<br>292<br>0.026	•••••<br>93<br>0.138	•••••<br>44<br>0.019	•••••<br>65<br>0.022	•••••<br>23<br>0.022	•••••<br>113<br>0.021	•••••<br>0.000	

Rina 2. Teil

Plate Flags:
Einzelfunktionsplatte

Ratio: < 0,8 neg.
≥ 0,8 - 1,1 GB
≥ 1,1 pos.

Analyzer Seriennummer: 202056

Leudeck 19.9.16

Dade Behring Marburg GmbH
Postfach 11 49
D-35001 Marburg

IgG

27.10.2016 12:47:56 Uhr

Plattenreport: Rohwerte

Platten-ID:	0011	Riegel:	1 - 11
BES Version:	V3.3	Validierung:	-
Analyzer:	202056		
Testname:	Photometrieren	Einheit:	-
Cut Off:	-	Valid.bereich	
Cut Off Formel:	-		
Wiederh.bereich:	-		
Blankkorrektur	-		
Niveau Korrektur	-		
Wellenlängen M/R:	450 nm / 650 nm		
Testkit Nr.:	-		
Platte definiert am:	27.10.2016 12:35 Uhr		

Zikka - Studie [signature]

!	Napfauffälligkeit		Ergebnis negativ
#	Keine Berechnung möglich	*	Ergebnis positiv
∞	Ergebnis zweifelhaft	?	Ergebnis in Wiederh.Ber.

IgG

	1	2	3	4	5	6	7	8	9	10	11	12
A	[illegible] 0.516	200 0.033	189 0.051	205 0.023	147 0.042	291 0.029	83 0.045	101 0.028	45 0.063	53 0.027	62 0.056	
B	pos. 0.938 1,217	153 0.047	264 0.214	219 0.018	302 0.096	166 0.028	73 0.031	84 0.040	35 0.033	43 0.029	42 0.052	
C	neg. 0.031 0,060	154 0.061	294 0.051	167 0.018	206 0.038	270 0.044	111 0.087	44 0.366	25 0.025	63 0.054	22 0.023	
D	172 0.018	175 0.022	224 0.035	160 0.110	278 0.031	152 0.015	108 0.050	15 0.018	06 0.017	72 0.042	168 0.641 1,251	
E	162 0.040	127 0.040	237 0,041	150 0.023	281 0.083	174 0.016	128 0.072	05 0.108	95 0.033	92 0.022	175 0.010	
F	221 0.040	161 0.028	182 0.027	148 0.035	274 0.025	142 0.031	14 0.080	94 0.027	85 0.144	03 0.023	-0.000	
G	222 0.037	196 0.103	141 0.017	168 0.024	234 0.017	151 0.050	130 0.329	84 0.057	75 0.026	13 0.041	0.000	
H	184 0.097	183 0.033	201 0.022	155 0.024	292 0.018	93 0.033	74 0.024	65 0.024	23 0.020	113 0.029	0.000	

WH ? Lieuz 2. Teil
WH

Plate Flags:
Einzelfunktionsplatte

Ratio: < 0,8 neg.
≥ 0,8 – 1,1 GW
≥ 1,1 pos.

Umhausen

GK

Dade Behring Marburg GmbH
Postfach 11 49
D-35001 Marburg

25.08.2016 12:45:35 Uhr

Plattenreport: Rohwerte

Platten-ID:	0006	Riegel:	1 - 9
BES Version:	V3.3	Validierung:	-
Analyzer:	202056		
Testname:	Photometrieren	Einheit:	-
Cut Off:	-	Valid.bereich	
Cut Off Formel:	-		
Wiederh.bereich:	- Zika-G		
Blankkorrektur	-		
Niveau Korrektur	- Euroimmun		
Wellenlängen M/R:	450 nm / 650 nm		
Testkit Nr.:	-		
Platte definiert am:	25.08.2016 12:38 Uhr		

!	Napfauffälligkeit	*	Ergebnis negativ
#	Keine Berechnung möglich	?	Ergebnis positiv
∞	Ergebnis zweifelhaft		Ergebnis in Wiederh.Ber.

Pos	ProbenID	Wert	Flag
A1	---- Kal 2	0.367 ✓	
B1	---- +	0.707	1,9 ✓
C1	---- –	0.023	0,06 ✓
D1	---- 20	0.032	-
E1	---- 23	0.025	-
F1	---- 25	0.024	-
G1	---- 27	0.017	-
H1	---- 28	0.020	-
A4	---- 58	0.016	-
B4	---- 59	0.021	-
C4	---- 61	0.012	-
D4	---- 66	0.017	-
E4	---- 68	0.052	-
F4	---- 70	0.036	-
G4	---- 71	0.019	-
H4	---- 72 *	0.019	-
A7	---- 85	0.022	-
B7	---- 86	0.134	-
C7	---- 87	0.012	-
D7	---- 88	0.060	-
E7	---- 104	0.041	-
F7	---- 105	0.030	-
G7	---- 107	0.023	-
H7	---- 108	0.022	-

Pos	ProbenID	Wert	Flag
A2	---- 31	0.034	-
B2	---- 33	0.018	-
C2	---- 34	0.064	-
D2	---- 35	0.024	-
E2	---- 38	0.031	-
F2	---- 39	0.027	-
G2	---- 40	0.021	-
H2	---- 41	0.024	-
A5	---- 73	0.024	-
B5	---- 74	0.012	-
C5	---- 77	0.080	-
D5	---- 78	0.018	-
E5	---- 79	0.028	-
F5	---- 80	0.021	-
G5	---- 84	0.036	-
H5	---- 85	0.169	-
A8	---- 110	0.031	-
B8	---- 111	0.024	-
C8	---- 112	0.019	-
D8	---- 113	0.031	-
E8	---- 114	0.033	-
F8	---- 116	0.040	-
G8	---- 117	0.023	-
H8	---- 118	0.026	-

Pos	ProbenID	Wert	Flag
A3	---- 42	0.022	-
B3	---- 43	0.018	-
C3	---- 44	0.032	-
D3	---- 48	0.124	-
E3	---- 49	0.019	-
F3	---- 51	0.019	-
G3	---- 53	0.029	-
H3	---- 54	0.037	-
A6	---- 87	0.014	-
B6	---- 88	0.021	-
C6	---- 89	0.011	-
D6	---- 90	0.020	-
E6	---- 91	0.025	-
F6	---- 92	0.021	-
G6	---- 93	0.038	-
H6	---- 94	0.023	-
A9	---- 119	0.018	-
B9	---- 123	0.018	-
C9	---- 124	0.024	-
D9	---- 125	0.031	-
E9	---- 130	0.031	-
F9	----	0.000	
G9	----	0.000	
H9	----	0.000	

Plate Flags:

Einzelfunktionsplatte

$$\text{Ratio} = \frac{\text{Ext. Probe}}{\text{Ext. Kal 2}}$$

neg < 0,8
gw ≥ 0,8 - < 1,1
pos ≥ 1,1

GK

Dade Behring Marburg GmbH
Postfach 11 49
D-35001 Marburg

25.08.2016 12:51:56 Uhr

Plattenreport: Rohwerte

Platten-ID:	0007	Riegel:	1 - 9
BES Version:	V3.3	Validierung:	-
Analyzer:	202056		
Testname:	Photometrieren	Einheit:	-
Cut Off:	-	Valid.bereich	
Cut Off Formel:	- Zika - M		
Wiederh.bereich:	-		
Blankkorrektur	- Euroimmun		
Niveau Korrektur	-		
Wellenlängen M/R:	450 nm / 650 nm		
Testkit Nr.:	-		
Platte definiert am:	25.08.2016 12:39 Uhr		

!	Napfauffälligkeit		Ergebnis negativ
#	Keine Berechnung möglich	*	Ergebnis positiv
∞	Ergebnis zweifelhaft	?	Ergebnis in Wiederh.Ber.

Pos	ProbenID	Wert	Flag	Pos	ProbenID	Wert	Flag	Pos	ProbenID	Wert	Flag
A1	— Kol	0.539	✓	A2	— 31	0.313	-	A3	— 42	0.026	-
B1	— +	1.173	2,18 ✓	B2	— 33	0.118	-	B3	— 43	0.076	-
C1	— –	0.039	0,07 ✓	C2	— 34	0.034	-	C3	— 44	0.114	-
D1	— 20	0.049	-	D2	— 35	0.055	-	D3	— 48	0.100	-
E1	— 23	0.098	-	E2	— 38	0.056	-	E3	— 49	0.055	-
F1	— 25	0.046	-	F2	— 39	0.060	-	F3	— 51	0.066	-
G1	— 27	0.063	-	G2	— 40	0.094	-	G3	— 53	0.062	-
H1	— 28	0.047	-	H2	— 41	0.081	-	H3	— 54	0.096	-
A4	— 58	0.021	-	A5	— 73	0.033	-	A6	— 87	0.053	-
B4	— 59	0.015	-	B5	— 74	0.020	-	B6	— 88	0.043	-
C4	— 61	0.029	-	C5	— 77	0.080	-	C6	— 89	0.020	-
D4	— 66	0.013	-	D5	— 78	0.016	-	D6	— 90	0.014	-
E4	— 68	0.044	-	E5	— 79	0.050	-	E6	— 91	0.025	-
F4	— 70	0.032	-	F5	— 80	0.047	-	F6	— 92	0.016	-
G4	— 71	0.038	-	G5	— 84	0.086	-	G6	— 93	0.023	-
H4	— 72	0.031	-	H5	— 85	0.059	-	H6	— 94	0.027	-
A7	— 95	0.031	-	A8	— 110	0.029	-	A9	— 119	0.011	-
B7	— 96	0.034	-	B8	— 111	0.017	-	B9	— 123	0.034	-
C7	— 97	0.036	-	C8	— 112	0.018	-	C9	— 124	0.050	-
D7	— 99	0.016	-	D8	— 113	0.027	-	D9	— 125	0.055	-
E7	— 104	0.026	-	E8	— 114	0.017	-	E9	— 130	0.014	-
F7	— 105	0.034	-	F8	— 116	0.070	-	F9	—	0.000	
G7	— 107	0.020	-	G8	— 117	0.049	-	G9	—	0.000	
H7	— 108	0.022	-	H8	— 118	0.031	-	H9	—	0.000	

Plate Flags:
Einzelfunktionsplatte

Ratio = Ext. Probe / Ext. Kol

Analyzer Seriennummer: 202056

Zirl / 03.11.16

Dade Behring Marburg GmbH
Postfach 11 49
D-35001 Marburg

IgM

8.11.16 11:51:12

Plattenreport: Rohwerte

Platten-ID:	0012	Riegel:	1 - 8
BES Version:	V3.3	Validierung:	-
Analyzer:	151549		
Testname:	Photometrieren	Einheit:	-
Cut Off:	-	Valid.bereich	
Cut Off Formel:	-		
Wiederh.bereich:	-		
Blankkorrektur	-		
Niveau Korrektur	-		
Wellenlängen M/R:	450 nm / 650 nm		
Testkit Nr.:	-		
Platte definiert am:	8.11.16 11:45		

Zikke - Studie [signature]

!	Napfauffälligkeit		Ergebnis negativ
#	Keine Berechnung möglich	*	Ergebnis positiv
∞	Ergebnis zweifelhaft	?	Ergebnis in Wiederh.Ber.

IgM

	1	2	3	4	5	6	7	8	9	10	11	12
A	Cal 0.545	017 0.036	031 0.036	051 0.032	067 0.026	079 0.036	097 0.022	112 0.103				
B	Pos. 1.277 1,343	020 0.023	034 0.163	057 0.091	068 0.029	084 0.048	100 0.043	113 0.024				
C	neg 0.031 0,056	021 0.094	035 0.018	058 0.020	071 0.189	085 0.049	101 0.058	115 0.029				
D	004 0.053	023 0.028	042 0.058	059 0.037	072 0.028	087 0.118	104 0.027	0.001				
E	007 0.035	025 0.038	044 0.069	060 0.019	074 0.047	090 0.050	106 0.072	-0.000				
F	009 0.020	026 0.234	046 0.054	062 0.019	075 0.020	091 0.033	107 0.032	0.00[illegible]				
G	013 0.021	029 0.062	047 0.017	064 0.058	076 0.055	094 0.022	108 0.076	-0.000				
H	015 0.051	030 0.091	050 0.025	065 0.014	077 0.031	095 0.019	110 0.057	0.000				

Plate Flags:
Einzelfunktionsplatte

Ratio: < 0,8 neg.
≥ 0,8 - 1,1 GW
≥ 1,1 pos.

Zirl / 03.11.16

Dade Behring Marburg GmbH
Postfach 11 49
D-35001 Marburg

IgG

8.11.16 12:01:57

Plattenreport: Rohwerte

Platten-ID:	0011	Riegel:	1 - 8
BES Version:	V3.3	Validierung:	-
Analyzer:	151549		
Testname:	Photometrieren	Einheit:	-
Cut Off:	-	Valid.bereich	
Cut Off Formel:	-		
Wiederh.bereich:	-		
Blankkorrektur	-		
Niveau Korrektur	-		
Wellenlängen M/R:	450 nm / 650 nm		
Testkit Nr.:	-		
Platte definiert am:	8.11.16 11:45		

Zika - Studie [signature]

!	Napfauffälligkeit		Ergebnis negativ
#	Keine Berechnung möglich	*	Ergebnis positiv
∞	Ergebnis zweifelhaft	?	Ergebnis in Wiederh.Ber.

IgG

	1	2	3	4	5	6	7	8	9	10	11	12
A	Cal. 0.418	017 0.032	031 0.038	051 0.027	067 0.080	079 0.029	097 0.049	112 0.072				
B	pos. 0.844 1,019	020 0.019	034 0.030	057 0.072	068 0.137	084 0.020	100 0.030	113 0.045				
C	neg. 0.029 0,069	021 0.040	035 0.016	058 0.037	071 0.054	085 0.022	101 0.039	115 0.030				
D	004 0.119	023 0.048	042 0.047	059 0.022	072 0.017	087 0.041	104 0.081	0.000				
E	007 0.041	025 0.043	044 0.031	060 0.019	074 0.035	090 0.026	106 0.228	-0.000				
F	009 0.027	026 0.029	046 0.017	062 0.018	075 0.085	091 0.033	107 0.046	-0.000				
G	013 0.017	029 0.026	047 0.019	064 0.033	076 0.029	094 0.061	108 0.052	-0.000				
H	015 0.035	030 0.034	050 0.030	065 0.042	077 0.022	095 0.021	110 0.020	0.000				

Plate Flags:
Einzelfunktionsplatte

Ratio: < 0,8 neg.
≥ 0,8 - 1,1 GW
≥ 1,1 pos.

Seefeld 21.10.16

Dade Behring Marburg GmbH
Postfach 11 49
D-35001 Marburg

IgM

3.11.16 12:40:28

Plattenreport: Rohwerte

Platten-ID:	0012	Riegel:	1 - 12
BES Version:	V3.3	Validierung:	-
Analyzer:	151549		
Testname:	Photometrieren	Einheit:	-
Cut Off:	-	Valid.bereich	
Cut Off Formel:	-		
Wiederh.bereich:	-		
Blankkorrektur	-		
Niveau Korrektur	-		
Wellenlängen M/R:	450 nm / 650 nm		
Testkit Nr.:	-		
Platte definiert am:	3.11.16 12:28		

Zilke-Studie [illegible]

!	Napfauffälligkeit		Ergebnis negativ
#	Keine Berechnung möglich	*	Ergebnis positiv
∞	Ergebnis zweifelhaft	?	Ergebnis in Wiederh.Ber.

IgM

	1	2	3	4	5	6	7	8	9	10	11	12
A	Cal. 0.405	095 0.025	010 0.040	078 0.024	060 0.027	043 0.076	058 0.019	028 0.021	019 0.036	004 0.024	109 0.014	061 0.035
B	pos. 1.048 2,581	087 0.013	018 0.036	073 0.015	063 0.030	039 0.021	056 0.092	029 0.024	026 0.520 1,280	013 0.016	030 0.028	051 0.023
C	neg. 0.023 0,056	092 0.014	099 0.017	081 0.015	061 0.019	049 0.028	037 0.031	041 0.033	016 0.031	022 0.027	081 0.024	0.005
D	106 0.017	084 0.020	077 0.017	082 0.019	067 0.039	052 0.299	038 0.016	005 0.017	031 0.017	008 0.021	091 0.019	0.001
E	105 0.014	094 0.034	075 0.017	079 0.028	066 0.017	050 0.016	030 0.043	012 0.019	033 0.030	003 0.020	002 0.025	-0.000
F	104 0.090	091 0.080	065 0.012	051 0.032	070 0.019	047 0.016	040 0.019	014 0.056	001 0.031	006 0.015	012 0.187	0.000
G	102 0.016	097 0.020	074 0.011	023 0.056	072 0.027	048 0.021	035 0.021	018 0.018	020 0.035	010 0.016	071 0.025	0.000
H	101 0.078	093 0.018	085 0.044	054 0.019	045 0.029	056 0.013	034 0.020	025 0.015	009 0.034	020 0.015	105 0.024	0.000

Randeck 2. Teil

Plate Flags:
Einzelfunktionsplatte

Ratio: < 0,8 neg.
≥ 0,8 - 1,1 GW
≥ 1,1 pos.

Seefeld 21.10.16

Dade Behring Marburg GmbH
Postfach 11 49
D-35001 Marburg

IgG

3.11.16 12:40:20

Plattenreport: Rohwerte

Platten-ID:	0011	Riegel:	1 - 12
BES Version:	V3.3	Validierung:	-
Analyzer:	151549		
Testname:	Photometrieren	Einheit:	-
Cut Off:	-	Valid.bereich	
Cut Off Formel:	-		
Wiederh.bereich:	-		
Blankkorrektur	-		
Niveau Korrektur	-		
Wellenlängen M/R:	450 nm / 650 nm		
Testkit Nr.:	-		
Platte definiert am:	3.11.16 12:28		

Zikta-Studie [signature]

!	Napfauffälligkeit		Ergebnis negativ
#	Keine Berechnung möglich	*	Ergebnis positiv
∞	Ergebnis zweifelhaft	?	Ergebnis in Wiederh.Ber.

	IgG	2	3	4	5	6	7	8	9	10	11	12
A	Cal. 0.415	095 0.022	010 0.019	078 0.022	060 0.021	043 0.023	058 0.022	018 0.018	019 0.035	004 0.036	109 0.016	061 0.019
B	pos. 0.856 2,062	087 0.027	018 0.018	073 0.030	063 0.123	039 0.033	036 0.019	029 0.016	026 0.061	013 0.063	030 0.069	051 0.030
C	neg. 0.024 0,057	092 0.032	099 0.021	081 0.019	061 0.025	049 0.024	037 0.016	041 0.032	016 0.016	022 0.032	081 0.051	0.002
D	106 0.015	084 0.046	077 0.021	082 0.025	067 0.027	052 0.035	038 0.017	005 0.035	031 0.022	008 0.017	011 0.015	0.000
E	103 0.013	094 0.024	075 0.025	079 0.020	066 0.025	050 0.016	030 0.027	012 0.022	033 0.026	003 0.021	002 0.054	-0.000
F	104 0.020	011 0.099	065 0.021	051 0.027	070 0.044	047 0.021	040 0.022	014 0.021	001 0.025	006 0.018	012 0.025	0.000
G	102 0.013	097 0.025	074 0.025	023 0.042	072 0.022	048 0.011	035 0.015	018 0.014	020 0.084	010 0.019	071 0.022	0.000
H	101 0.045	093 0.022	085 0.027	054 0.017	045 0.026	056 0.013	034 0.014	025 0.021	009 0.021	020 0.018	105 0.028	0.000

Ausdruck 2. Teil

Plate Flags:

Einzelfunktionsplatte

Ratio: < 0,8 neg.
≥ 0,8 – 1,1 GW
≥ 1,1 pos.

Neustift 19.10.16

Dade Behring Marburg GmbH
Postfach 11 49
D-35001 Marburg

IgG

02.11.2016 11:59:18 Uhr

Plattenreport: Rohwerte

Platten-ID:	0011	Riegel:	1 - 7
BES Version:	V3.3	Validierung:	-
Analyzer:	202056		
Testname:	Photometrieren	Einheit:	-
Cut Off:	-	Valid.bereich	
Cut Off Formel:	-		
Wiederh.bereich:	-		
Blankkorrektur	-		
Niveau Korrektur	-		
Wellenlängen M/R:	450 nm / 650 nm		
Testkit Nr.:	-		
Platte definiert am:	02.11.2016 11:47 Uhr		

Zika - Studie [signature]

!	Napfauffälligkeit	*	Ergebnis negativ
#	Keine Berechnung möglich	?	Ergebnis positiv
∞	Ergebnis zweifelhaft		Ergebnis in Wiederh.Ber.

	1 (IgG)	2	3	4	5	6	7	8	9	10	11	12
A	••••• cal. 0.486	••••• 098 0.061	••••• 113 0.025	••••• 052 0.169	••••• 069 0.348	••••• 046 0.028	••••• 009 0.029					
B	••••• pos. 0.901 1,855	••••• 106 0.024	••••• 068 0.024	••••• 053 0.035	••••• 070 0.019	••••• 045 0.040	••••• 008 0.024					
C	••••• neg. 0.033 0,057	••••• 114 0.057	••••• 079 0.110	••••• 054 0.057	••••• 029 0.015	••••• 041 0.015	••••• 005 0.014					
D	••••• 131 0.024	••••• 116 0.022	••••• 081 0.079	••••• 057 0.018	••••• 030 0.037	••••• 050 0.018	••••• 003 0.025					
E	••••• 126 0.040	••••• 120 0.045	••••• 086 0.035	••••• 051 0.084	••••• 033 0.064	••••• 026 0.072	••••• 002 0.095					
F	••••• 091 0.045	••••• 124 0.017	••••• 097 0.020	••••• 059 0.017	••••• 036 0.019	••••• 025 0.036	••••• 001 0.052					
G	••••• 092 0.034	••••• 071 0.034	••••• 107 0.038	••••• 055 0.024	••••• 037 0.025	••••• 017 0.148	~~••••• 0.000~~					
H	••••• 094 0.097	••••• 073 0.036	••••• 112 0.046	••••• 062 0.042	••••• 047 0.014	••••• 022 0.029	~~••••• 0.000~~					

Plate Flags:
Einzelfunktionsplatte

Ratio: < 0,8 neg.
≥ 0,8 - 1,1 GW
≥ 1,1 pos.

Neustift 19.10.16

Dade Behring Marburg GmbH
Postfach 11 49
D-35001 Marburg

IgM

02.11.2016 11:59:29 Uhr

Plattenreport: Rohwerte

Platten-ID:	0012	Riegel:	1 - 7
BES Version:	V3.3	Validierung:	-
Analyzer:	202056		
Testname:	Photometrieren	Einheit:	-
Cut Off:	-	Valid.bereich	
Cut Off Formel:	-		
Wiederh.bereich:	-		
Blankkorrektur	-		
Niveau Korrektur	-		
Wellenlängen M/R:	450 nm / 650 nm		
Testkit Nr.:	-		
Platte definiert am:	02.11.2016 11:47 Uhr		

Zika - Studie [signature]

!	Napfauffälligkeit		Ergebnis negativ
#	Keine Berechnung möglich	*	Ergebnis positiv
∞	Ergebnis zweifelhaft	?	Ergebnis in Wiederh.Ber.

IgM

	1	2	3	4	5	6	7	8	9	10	11	12
A	●●●●● Cal. 0.556	●●●●● 098 0.047	●●●●● 113 0.059	●●●●● 052 0.043	●●●●● 069 0.030	●●●●● 046 0.016	●●●●● 009 0.037					
B	●●●●● pos. 1.336 2,402	●●●●● 106 0.053	●●●●● 068 0.021	●●●●● 053 0.038	●●●●● 070 0.036	●●●●● 045 0.024	●●●●● 008 0.015					
C	●●●●● neg. 0.029 0,052	●●●●● 114 0.044	●●●●● 079 0.026	●●●●● 054 0.015	●●●●● 029 0.093	●●●●● 041 0.015	●●●●● 005 0.076					
D	●●●●● 131 0.030	●●●●● 116 0.052	●●●●● 081 0.048	●●●●● 057 0.013	●●●●● 030 0.068	●●●●● 050 0.019	●●●●● 003 0.053					
E	●●●●● 126 0.069	●●●●● 120 0.116	●●●●● 086 0.016	●●●●● 051 0.036	●●●●● 033 0.083	●●●●● 026 0.067	●●●●● 002 0.014					
F	●●●●● 091 0.076	●●●●● 124 0.017	●●●●● 097 0.018	●●●●● 059 0.025	●●●●● 036 0.033	●●●●● 025 0.087	●●●●● 001 0.027					
G	●●●●● 092 0.016	●●●●● 071 0.030	●●●●● 107 0.089	●●●●● 055 0.039	●●●●● 037 0.032	●●●●● 017 0.023	●●●●● 0.000 (crossed out)					
H	●●●●● 094 0.029	●●●●● 073 0.024	●●●●● 112 0.038	●●●●● 062 0.052	●●●●● 047 0.011	●●●●● 022 0.038	●●●●● 0.000 (crossed out)					

Plate Flags:
Einzelfunktionsplatte

Ratio: < 0,8 neg.
≥ 0,8 - 1,1 GW
≥ 1,1 pos.

Igb 1.10. 16

Dade Behring Marburg GmbH
Postfach 11 49
D-35001 Marburg

17.10.2016 13:23:08 Uhr

Plattenreport: Rohwerte

IGG

Platten-ID:	0011	Riegel:	1 - 2
BES Version:	V3.3	Validierung:	-
Analyzer:	202056		
Testname:	Photometrieren	Einheit:	-
Cut Off:	-	Valid.bereich	
Cut Off Formel:	-		
Wiederh.bereich:	-	Zikka Elisa IgG	
Blankkorrektur	-		
Niveau Korrektur	-		
Wellenlängen M/R:	450 nm / 650 nm		
Testkit Nr.:	-		
Platte definiert am:	17.10.2016 13:17 Uhr		

!	Napfauffälligkeit		Ergebnis negativ
#	Keine Berechnung möglich	*	Ergebnis positiv
∞	Ergebnis zweifelhaft	?	Ergebnis in Wiederh.Ber.

IgG

	1	2	3	4	5	6	7	8	9	10	11	12
A	Cal. 0.533	007 0.042 0,223										
B	pos. 1.063 1,097	011 0.032 0,060										
C	ldg. 0.036 0,067	0013 0.049 0,091										
D	001 0.042 0,078	0015 0.044 0,082										
E	003 0.023 0,043	0016 0.093 0,174										
F	004 0.022 0,041	-0.000										
G	005 0.081 0,151	-0.000										
H	006 0.067 0,125	-0.000										

Plate Flags:
Einzelfunktionsplatte

Ratio: < 0,8 neg.
≥ 0,8-1,1 grenzwertig
≥ 1,1 pos.

Iglis 1.10.16

Dade Behring Marburg GmbH
Postfach 11 49
D-35001 Marburg

17.10.2016 13:23:01 Uhr

IGM

Plattenreport: Rohwerte

Platten-ID:	0012	Riegel:	1 - 2
BES Version:	V3.3	Validierung:	-
Analyzer:	202056		
Testname:	Photometrieren	Einheit:	-
Cut Off:	-	Valid.bereich	
Cut Off Formel:	-		
Wiederh.bereich:	-		
Blankkorrektur	-		
Niveau Korrektur	-		
Wellenlängen M/R:	450 nm / 650 nm		
Testkit Nr.:	-		
Platte definiert am:	17.10.2016 13:18 Uhr		

Zikke Elisa IgM

[signature]

!	Napfauffälligkeit		Ergebnis negativ
#	Keine Berechnung möglich	*	Ergebnis positiv
∞	Ergebnis zweifelhaft	?	Ergebnis in Wiederh.Ber.

	1 (IgM)	2	3	4	5	6	7	8	9	10	11	12
A	••••• Cal. 0.549	••••• 007 0.030 0,054										
B	••••• pos. 1.329 2,420	••••• 011 0.020 0,036										
C	••••• neg. 0.034 0,061	••••• 0013 0.042 0,[illegible]6										
D	••••• 001 0.032 0,058	••••• 0015 0.249 0,453										
E	••••• 003 0.041 0,074	••••• 0016 0.035 0,063										
F	••••• 004 0.046 0,083	••••• -0.000										
G	••••• 005 0.090 0,163	••••• -0.000										
H	••••• 006 0.020 0,036	••••• -0.000										

Plate Flags:
Einzelfunktionsplatte

Ratio: < 0,8 neg.
≥ 0,8-1,1 grenzwertig
≥ 1,1 pos.
