# Supplementary material for: No molecular or serological evidence of Zikavirus infection among healthy blood donors living in or travelling to regions where Aedes albopictus circulates
Source: PLoS One. 2017 May 24;12(5):e0178175. doi: 10.1371/journal.pone.0178175 (PMC5443526; doi:10.1371/journal.pone.0178175)
Supplement: S1 Table — (PDF) [file pone.0178175.s006.pdf]

S1. Table . A raw data on country and/or exact destination visited by participants between April and October 2016

		Country visited								
		no history of travel	Italy	Croatia	Greece	Spain	France	Others	More than one destination	Total
Exact destination		411	1	0	0	0	0	0	0	412
	Ägypten	0	0	0	0	0	0	2	0	2
	Antalia_Krk	0	0	0	0	0	0	1	0	1
	Antalya_Side	0	0	0	0	0	0	1	0	1
	Äqypten_Hurghada	0	0	0	0	0	0	1	0	1
	Assisi	0	1	0	0	0	0	0	0	1
	Barcelona	0	0	0	0	3	0	0	0	3
	Barcelona;Meran	0	0	0	0	0	0	0	1	1
	Bibione	0	1	0	0	0	0	0	0	1
	Bosnien	0	0	0	0	0	0	1	0	1
	Bosnien_Derventa_Italien_Bozen	0	0	0	0	0	0	1	0	1
	Bosnien_Slowenien_Italien_Kroatien	0	0	0	0	0	0	1	0	1
	Brenner	0	1	0	0	0	0	0	0	1
	Brenta_Dolomiten_Trient	0	1	0	0	0	0	0	0	1
	Bruneck	0	1	0	0	0	0	0	0	1
	Budva	0	0	0	0	0	0	1	0	1
	Bulgarien_Sonnenstrand	0	0	0	0	0	0	1	0	1
	Caldonazzo	0	1	0	0	0	0	0	0	1
	Caldonazzo_Rom_Ajjaro_Barcelona_Marseilles	0	0	0	0	0	0	0	1	1
	Cavallino	0	1	0	0	0	0	0	0	1
	Cesenatico	0	1	0	0	0	0	0	0	1
	Chalkidiki	0	0	0	1	0	0	0	0	1

	Chioggia	0	1	0	0	0	0	0	0	1
	Elba	0	1	0	0	0	0	0	0	1
	Frankreich	0	0	0	0	0	1	0	0	1
	Frankreich_Cote'd_Azur_Italien_Gardasee	0	0	0	0	0	1	0	0	1
	Frankreich_Grenoble	0	0	0	0	0	1	0	0	1
	Frankreich_Korsika	0	0	0	0	0	1	0	0	1
	Frankreich_Lourdes	0	0	0	0	0	1	0	0	1
	Frankreich_Paris	0	0	0	0	0	1	0	0	1
	Frankreich_Sophia_Antipolis_Nantes	0	0	0	0	0	1	0	0	1
	Frankreich;Italien;Spanien	0	0	0	0	0	0	0	1	1
	Gardasee	0	6	0	0	0	0	0	0	6
	Gardasee_Cavallino	0	1	0	0	0	0	0	0	1
	Gardasee_Frankreich_Chamonix	0	0	0	0	0	0	0	1	1
	Gardasee_Griechenland_Epiros	0	0	0	0	0	0	0	1	1
	Gardasee_Kreta	0	0	0	0	0	0	0	1	1
	Gardasee_Krk_Mallorca	0	0	0	0	0	0	0	1	1
	Gardasee_Mallorca_Lanzerotte	0	0	0	0	0	0	0	1	1
	Gardasee_Montpellier_Barcelona_Lissabon	0	0	0	0	0	0	0	1	1

	Gardasee_Sirmione	0	1	0	0	0	0	0	0	1
	Gardasee_Toscana/_St.Tropez/_Mallorca_Alba_Piemont	0	0	0	0	0	0	0	1	1
	Genua_Monaco_Valencia_Barcelona_Korsika_Cannes	0	0	0	0	0	0	0	1	1
	Grado	0	1	0	0	0	0	0	0	1
	Gran_Canaria	0	0	0	0	1	0	0	0	1
	Gran_Canaria_Mallorca	0	0	0	0	1	0	0	0	1
	Griechenland	0	0	0	1	0	0	0	0	1
	Griechenland_Athen	0	0	0	1	0	0	0	0	1
	Griechenland_Chalkidiki	0	0	0	2	0	0	0	0	2
	Griechenland_Chalkidiki, Italien_Jesolo	0	0	0	1	0	0	0	0	1
	Griechenland_Italien	0	0	0	1	0	0	0	1	2
	Griechenland_Kalymnos	0	0	0	3	0	0	0	0	3
	Griechenland_Kefalonia	0	0	0	1	0	0	0	0	1
	Griechenland_Korfu	0	0	0	1	0	0	0	0	1
	Griechenland_Korfu_Italien_Savona_Frankreich_Spanien_Kroatien_Slowenien	0	0	0	1	0	0	0	0	1

	Griechenland_Korfu_Kroatien_Opatija	0	0	0	1	0	0	0	0	1
	Griechenland_Kos	0	0	0	3	0	0	0	0	3
	Griechenland_Kos_Tigaki	0	0	0	1	0	0	0	0	1
	Griechenland_Kreta;Türkei_Antolia	0	0	0	0	0	0	0	1	1
	Griechenland_Lefkas_Agios_Nikitas	0	0	0	1	0	0	0	0	1
	Griechenland_Parga	0	0	0	1	0	0	0	0	1
	Griechenland_Rhodos	0	0	0	8	0	0	0	0	8
	Griechenland_Santorin_Milos_Sifuos_Folegandros	0	0	0	1	0	0	0	0	1
	Griechenland_Thessaloniki	0	0	0	1	0	0	0	0	1
	Griechenland_Vrachos_Beach	0	0	0	1	0	0	0	0	1
	Iatlien_Jesolo_Frankreich_Paris	0	1	0	0	0	0	0	0	1
	Iatlien_Rimini	0	1	0	0	0	0	0	0	1
	Ibiza_Mallorca_Sizilien	0	0	0	0	0	0	0	1	1
	Istrien	0	0	3	0	0	0	0	0	3
	Italie_Sizilien	0	1	0	0	0	0	0	0	1
	Italien	0	10	0	0	0	0	0	0	10
	Italien_Abano_Padua	0	1	0	0	0	0	0	0	1
	Italien_Adria	0	1	0	0	0	0	0	0	1

	Italien_Adria_Gargano_Umbrien	0	1	0	0	0	0	0	0	1
	Italien_Alta_Badia	0	1	0	0	0	0	0	0	1
	Italien_Am_Ritten	0	1	0	0	0	0	0	0	1
	Italien_Apulien	0	2	0	0	0	0	0	0	2
	Italien_Assisi_Florenz	0	1	0	0	0	0	0	0	1
	Italien_Bardolino	0	2	0	0	0	0	0	0	2
	Italien_Bibione	0	8	0	0	0	0	0	0	8
	Italien_Bibione_Gardasee_Toskana_Marina_di_Bibbona_Kroatien_Rovinj	0	1	0	0	0	0	0	0	1
	Italien_Bozen	0	5	0	0	0	0	0	0	5
	Italien_Brenner	0	1	0	0	0	0	0	0	1
	Italien_Bruneck	0	1	0	0	0	0	0	0	1
	Italien_Calabrien	0	1	0	0	0	0	0	0	1
	Italien_Calabrien_Kroatien_Hvar	0	0	0	0	0	0	0	1	1
	Italien_Caorle	0	5	0	0	0	0	0	1	6
	Italien_Caorle_Kroatien_Rabac	0	1	0	0	0	0	0	0	1
	Italien_Caorle_Obere_Adria	0	1	0	0	0	0	0	0	1
	Italien_Cavalino	0	1	0	0	0	0	0	0	1

	Italien_Cavallino	0	7	0	0	0	0	0	0	7
	Italien_Cervia	0	1	0	0	0	0	0	0	1
	Italien_Chioggia	0	1	0	0	0	0	0	0	1
	Italien_Cinque_Terre	0	2	0	0	0	0	0	0	2
	Italien_Cinque_Terre;Griechenland_Korfu	0	0	0	0	0	0	0	1	1
	Italien_Desenzano	0	1	0	0	0	0	0	0	1
	Italien_Durchreise_Frankreich_Piermont_Seealpen_Spanien_Andalusien_Alicante_Valencia	0	0	0	0	0	0	0	1	1
	Italien_Elba	0	3	0	0	0	0	0	0	3
	Italien_Ferrara	0	1	0	0	0	0	0	0	1
	Italien_Florenz	0	1	0	0	0	0	0	0	1
	Italien_Follonica	0	1	0	0	0	0	0	0	1
	Italien_Frankreich_Cdmar	0	0	0	0	0	0	0	1	1
	Italien_Frankreich_Spanien	0	0	0	0	0	0	0	1	1
	Italien_Frankreich_Spanien(Kreuzfahrt)	0	0	0	0	0	0	0	1	1
	Italien_Friaul_Griechenland_Thassos_Kroatien_Krk	0	0	0	0	0	0	0	1	1

	Italien_Gardasee	0	41	0	0	0	0	0	0	41
	Italien_Gardasee_Bardolino	0	2	0	0	0	0	0	0	2
	Italien_Gardasee_Cavallino	0	1	0	0	0	0	0	0	1
	Italien_Gardasee_Florenz	0	1	0	0	0	0	0	0	1
	Italien_Gardasee_Grado	0	1	0	0	0	0	0	0	1
	Italien_Gardasee_Griechenland_Kalymnos	0	0	0	0	0	0	0	1	1
	Italien_Gardasee_Jesolo	0	1	0	0	0	0	0	0	1
	Italien_Gardasee_Lido_del_Nazioni	0	1	0	0	0	0	0	0	1
	Italien_Gardasee_Limone	0	1	0	0	0	0	0	0	1
	Italien_Gardasee_Malcesine	0	1	0	0	0	0	0	0	1
	Italien_Gardasee_Rimini	0	1	0	0	0	0	0	0	1
	Italien_Gardasee_Riva_del_Garda	0	1	0	0	0	0	0	0	1
	Italien_Gardasee_Rom	0	1	0	0	0	0	0	0	1
	Italien_Gardasee_Sirmione	0	1	0	0	0	0	0	0	1
	Italien_Gardasee_Torbole	0	1	0	0	0	0	0	0	1

	Italien_Gardasee_Tremosine	0	1	0	0	0	0	0	0	1
	Italien_Gardasee_Verona	0	1	0	0	0	0	0	0	1
	Italien_Gardasee,Spanien_Kanaren	0	0	0	0	0	0	0	1	1
	Italien_Gardasee;Barcelona	0	0	0	0	0	0	0	1	1
	Italien_Gardasee;Kroatien_Istrien	0	0	0	0	0	0	0	1	1
	Italien_Gardasee;Kroatien_Rovinj	0	0	0	0	0	0	0	1	1
	Italien_Gemona	0	1	0	0	0	0	0	0	1
	Italien_Grado	0	2	0	0	0	0	0	0	2
	Italien_Griechenland	0	0	0	0	0	0	0	1	1
	Italien_Jesolo	0	18	0	0	0	0	0	0	18
	Italien_Jesolo_Cavalino	0	1	0	0	0	0	0	0	1
	Italien_Jesolo_Kroatien_Vrsar	0	0	0	0	0	0	0	1	1
	Italien_Kalabrien	0	1	0	0	0	0	0	0	1
	Italien_Kalterer_See	0	1	0	0	0	0	0	0	1
	Italien_Kampanien_Sorrent_Neapel_Toskana_Sorano	0	1	0	0	0	0	0	0	1
	Italien_Kroatien	0	1	0	0	0	0	0	0	1

	Italien_La_Spezia_Lago_di_Garda_Rimini_Elba	0	1	0	0	0	0	0	0	1
	Italien_Lago_Maggiore	0	1	0	0	0	0	0	0	1
	Italien_Lazise	0	1	0	0	0	0	0	0	1
	Italien_Lazise_Gardasee	0	1	0	0	0	0	0	0	1
	Italien_Lazise_Rovino	0	1	0	0	0	0	0	0	1
	Italien_Lazisse_Triest_Venedig_Kroatien_medulin	0	0	0	0	0	0	0	1	1
	Italien_Lido_di_Jesolo	0	1	0	0	0	0	0	0	1
	Italien_Lignano	0	8	0	0	0	0	0	0	8
	Italien_Lignano_Bibione	0	1	0	0	0	0	0	0	1
	Italien_Madrid	0	1	0	0	0	0	0	0	1
	Italien_Mailand	0	1	0	0	0	0	0	0	1
	Italien_Marocco	0	1	0	0	0	0	0	0	1
	Italien_Meran	0	1	0	0	0	0	0	0	1
	Italien_Meran_Gardasee	0	1	0	0	0	0	0	0	1
	Italien_Modena	0	2	0	0	0	0	0	0	2
	Italien_Montegrotto	0	1	0	0	0	0	0	0	1
	Italien_Montegrotto_Kroatien_Zadar_Split_Novigrad	0	0	0	0	0	0	0	1	1

	Italien_Nizza_Gardasee	0	1	0	0	0	0	0	0	1
	Italien_Padua_Assisi_Rimini_Bologna	0	1	0	0	0	0	0	0	1
	Italien_Pardolino_Gardasee	0	1	0	0	0	0	0	0	1
	Italien_Pesaro	0	1	0	0	0	0	0	0	1
	Italien_Peschiera	0	1	0	0	0	0	0	0	1
	Italien_Pesciera	0	1	0	0	0	0	0	0	1
	Italien_Pisa	0	1	0	0	0	0	0	0	1
	Italien_Ravenna	0	1	0	0	0	0	0	0	1
	Italien_Rimini	0	1	0	0	0	0	0	0	1
	Italien_Ritten	0	1	0	0	0	0	0	0	1
	Italien_Rom	0	4	0	0	0	0	0	0	4
	Italien_Rom_Griechenland_Epiros	0	0	0	0	0	0	0	1	1
	Italien_Rom_Jesolo_Rimini_San_Marino	0	1	0	0	0	0	0	0	1
	Italien_Rom_Spanien_Mallorca_Kroatien_Istrien	0	0	0	0	0	0	0	1	1
	Italien_San_Remo_Triest_Frankreich_Cote_d'Azur	0	0	0	0	0	0	0	1	1
	Italien_Sardinien	0	5	0	0	0	0	0	0	5
	Italien_Sardinien_Neapel	0	1	0	0	0	0	0	0	1

	Italien_Savona,Frankreich_Marseille	0	0	0	0	0	0	0	1	1
	Italien_Schio	0	1	0	0	0	0	0	0	1
	Italien_Sizilien_Palermo_Cefalu	0	1	0	0	0	0	0	0	1
	Italien_Sizilien_Südspanien	0	0	0	0	0	0	0	1	1
	Italien_Südtirol	0	8	0	0	0	0	0	0	8
	Italien_Südtirol_Kroatien_Split	0	0	0	0	0	0	0	1	1
	Italien_Südtirol_Meran	0	1	0	0	0	0	0	0	1
	Italien_Torbole	0	1	0	0	0	0	0	0	1
	Italien_Toscana	0	2	0	0	0	0	0	0	2
	Italien_Toscana_Capri_Brenner;Frankreich_Paris	0	0	0	0	0	0	0	1	1
	Italien_Toscana_Frankreich_im_Westen	0	0	0	0	0	0	0	1	1
	Italien_Toscana_Gardasee;Frankreich_Dijon	0	0	0	0	0	0	0	1	1
	Italien_Toskana	0	7	0	0	0	0	0	0	7
	Italien_Trentino	0	3	0	0	0	0	0	0	3
	Italien_Trient	0	2	0	0	0	0	0	0	2
	Italien_Triest	0	4	0	0	0	0	0	0	4
	Italien_Tropea	0	1	0	0	0	0	0	0	1
	Italien_Udine	0	1	0	0	0	0	0	0	1

	Italien_Umbrien_Marren	0	1	0	0	0	0	0	0	1
	Italien_Venedig	0	4	0	0	0	0	0	0	4
	Italien_Venedig_Kroatien_Porec	0	0	0	0	0	0	0	1	1
	Italien_Venedig_Spanien_Fuerteventura	0	0	0	0	0	0	0	1	1
	Italien_Venetien	0	1	0	0	0	0	0	0	1
	Italien_Verona	0	1	0	0	0	0	0	0	1
	Italien_Via_Reggio	0	1	0	0	0	0	0	0	1
	Italien_Viserba	0	1	0	0	0	0	0	0	1
	Italien-Caorle	0	1	0	0	0	0	0	0	1
	Itallien_Gardasee	0	1	0	0	0	0	0	0	1
	Itallien_Rimini	0	1	0	0	0	0	0	0	1
	Jesolo	0	6	0	0	0	0	0	0	6
	Jesolo_Gardasee	0	1	0	0	0	0	0	0	1
	Jesolo_Venedig	0	1	0	0	0	0	0	0	1
	Kalabrien	0	1	0	0	0	0	0	0	1
	Kalterer_See	0	1	0	0	0	0	0	0	1
	Korfu	0	0	0	1	0	0	0	0	1
	Korsika_Italien	0	0	0	0	0	0	0	1	1
	Kos	0	0	0	1	0	0	0	0	1
	Kotor_Bari	0	0	0	0	0	0	0	1	1
	Kreta	0	0	0	5	0	0	0	0	5

	Kreuzfahrt_Le_Havre_Vigo_Barcelona_Valencia_Savona	0	0	0	0	0	0	0	1	1
	Kreuzfahrt_Mittelmeer	0	0	0	0	0	0	0	1	1
	Kroaien_Istrien_Split_Bosnien_Sarajevo_Slowenien_Ljubljana_Spanien_Bilbao_San_Sebastian_Italien	0	0	0	0	0	0	0	1	1
	Kroatein_	0	0	1	0	0	0	0	0	1
	Kroatien	0	0	7	0	0	0	0	0	7
	Kroatien_Brac	0	0	1	0	0	0	0	0	1
	Kroatien_Ceek_Venezia_Rab	0	0	0	0	0	0	0	1	1
	Kroatien_Cres	0	0	1	0	0	0	0	0	1
	Kroatien_Dubrovnik_Italien_Aostatal	0	0	0	0	0	0	0	1	1
	Kroatien_Insel_um_Split_Slowenien_Mazedonien_Montenegro	0	0	0	0	0	0	0	1	1
	Kroatien_Istrien	0	0	4	0	0	0	0	0	4
	Kroatien_Istrien_Novigrad	0	0	1	0	0	0	0	0	1
	Kroatien_Istrien_Rovinj	0	0	1	0	0	0	0	0	1
	Kroatien_Istrien_Umag	0	0	1	0	0	0	0	0	1
	Kroatien_Italien	0	0	0	0	0	0	0	1	1

	Kroatien_Krk	0	0	9	0	0	0	0	0	9
	Kroatien_Krk_Cres_Italien_Rimini	0	0	0	0	0	0	0	1	1
	Kroatien_Krk_Malinska	0	0	1	0	0	0	0	0	1
	Kroatien_Krk_Njivice	0	0	1	0	0	0	0	0	1
	Kroatien_Krk_Pula	0	0	1	0	0	0	0	0	1
	Kroatien_Lanezza	0	0	1	0	0	0	0	0	1
	Kroatien_Lanterna	0	0	1	0	0	0	0	0	1
	Kroatien_Mali_Losinj	0	0	1	0	0	0	0	0	1
	Kroatien_Mallorca	0	0	0	0	0	0	0	1	1
	Kroatien_Mareda	0	0	1	0	0	0	0	0	1
	Kroatien_Medulin	0	0	1	0	0	0	0	0	1
	Kroatien_Opatija	0	0	2	0	0	0	0	0	2
	Kroatien_Pag	0	0	2	0	0	0	0	0	2
	Kroatien_Parec	0	0	1	0	0	0	0	0	1
	Kroatien_Porec	0	0	16	0	0	0	0	0	16
	Kroatien_Porec_Funtana	0	0	1	0	0	0	0	0	1
	Kroatien_Porec_Italien_Lignano	0	0	0	0	0	0	0	1	1
	Kroatien_Pozega	0	0	1	0	0	0	0	0	1
	Kroatien_Pula	0	0	3	0	0	0	0	0	3
	Kroatien_Rab	0	0	2	0	0	0	0	0	2

	Kroatien_Rabac	0	0	2	0	0	0	0	0	2
	Kroatien_Rorinj	0	0	3	0	0	0	0	0	3
	Kroatien_Rovinj	0	0	2	0	0	0	0	0	2
	Kroatien_Senj	0	0	1	0	0	0	0	0	1
	Kroatien_Simuni	0	0	1	0	0	0	0	0	1
	Kroatien_Split_Solta_Sinj_Livno_Medugorje	0	0	1	0	0	0	0	0	1
	Kroatien_Süden;Bosnien_West	0	0	1	0	0	0	0	0	1
	Kroatien_Umag	0	0	5	0	0	0	0	0	5
	Kroatien_Vrsar	0	0	4	0	0	0	0	0	4
	Kroatien_Zadar	0	0	2	0	0	0	0	0	2
	Kurtatsch_Monaco	0	0	0	0	0	1	0	0	1
	Lefkas	0	0	0	2	0	0	0	0	2
	Madeira	0	0	0	0	0	0	1	0	1
	Madrid	0	0	0	0	1	0	0	0	1
	Malaga	0	0	0	0	1	0	0	0	1
	Mallorca	0	0	0	0	12	0	0	0	12
	Mallorca_Ibira_Barcelona_Neapel_Savona	0	0	0	0	1	0	0	0	1
	Mallorca_Jesolo	0	0	0	0	1	0	0	0	1
	Mallorca_Malta	0	0	0	0	0	0	0	1	1
	Mallorca_Paguera	0	0	0	0	1	0	0	0	1
	Mallorca;Portugal_Algarve	0	0	0	0	0	0	0	1	1

	Malta	0	0	0	0	0	0	1	0	1
	Malta;Italien_Meran_Gardasee_Lazise	0	0	0	0	0	0	0	1	1
	Meran	0	7	0	0	0	0	0	0	7
	Monerba	0	1	0	0	0	0	0	0	1
	Nizza	0	0	0	0	0	2	0	0	2
	Norditalien	0	2	0	0	0	0	0	0	2
	Otok_Cres	0	0	1	0	0	0	0	0	1
	Peschiera	0	1	0	0	0	0	0	0	1
	Piran	0	0	1	0	0	0	0	0	1
	Portugal	0	0	0	0	0	0	1	0	1
	Portugal_Lissabon	0	0	0	0	0	0	1	0	1
	Provence	0	1	0	0	0	0	0	0	1
	Pula	0	0	1	0	0	0	0	0	1
	Pustertal	0	1	0	0	0	0	0	0	1
	Pyrenäen	0	0	0	0	0	0	0	1	1
	Rimini	0	2	0	0	0	0	0	0	2
	Rimini_Bellaria	0	1	0	0	0	0	0	0	1
	San_Benetteo_Teltronto	0	1	0	0	0	0	0	0	1
	Santanyi	0	0	0	0	1	0	0	0	1
	Santiago_de_compostela	0	0	0	0	0	1	0	0	1
	Sardinien	0	1	0	0	0	0	0	0	1
	Savona_Lissabon_Cadiz_Rom_Casablanca_Alicante_	0	0	0	0	0	0	1	0	1
	Schweiz_Chiassia	0	0	0	0	0	0	0	1	1
	Selce	0	0	2	0	0	0	0	0	2
	Serbien_Subotica	0	0	0	0	0	0	1	0	1
	Slowenien_Dobrna	0	0	0	0	0	0	1	0	1
	Spanien	0	0	0	0	4	0	0	0	4

	Spanien_Barcelona	0	0	0	0	3	0	0	0	3
	Spanien_Frankreich_Italien	0	0	0	0	0	0	0	1	1
	Spanien_Fuerteventura	0	0	0	0	1	0	0	0	1
	Spanien_Gran_Canaria	0	0	0	0	1	0	0	0	1
	Spanien_Granada_Italien;Frankreich_Paris	0	0	0	0	0	0	0	1	1
	Spanien_La_Palma_Portugal_Lissabon	0	0	0	0	1	0	0	0	1
	Spanien_Lanzarote	0	0	0	0	1	0	0	0	1
	Spanien_Marbella_Sevillia_Granada_Malaga	0	0	0	0	1	0	0	0	1
	Spanien_Menorca	0	0	0	0	1	0	0	0	1
	Spanien_Sevilla_Frankreich_Normandie_Bretagne	0	0	0	0	0	0	0	1	1
	Split;Garda	0	1	0	0	0	0	0	0	1
	Sterzing	0	1	0	0	0	0	0	0	1
	Südfrankreich_Spanien	0	0	0	0	0	0	0	1	1
	Südtirol	0	1	0	0	0	0	0	0	1
	Südtirol_Kalterer_See	0	1	0	0	0	0	0	0	1
	Südtirol_Spanien_Nerja	0	0	0	0	0	0	0	1	1
	Südtirol_Sterzing	0	1	0	0	0	0	0	0	1
	Südtirol;Kreta	0	1	0	0	0	0	0	0	1

	Toblade	0	1	0	0	0	0	0	0	1
	Toskana	0	1	0	0	0	0	0	0	1
	Toskana_Jesolo	0	1	0	0	0	0	0	0	1
	Trient	0	1	0	0	0	0	0	0	1
	Trient_Chamonix_	0	1	0	0	0	0	0	0	1
	Tschechien_Krumau_Slowenien_Italien	0	0	0	0	0	0	0	1	1
	Türkei	0	0	0	0	0	0	1	0	1
	Türkei_Alanya;Italien_Desenzano_Gardasee	0	0	0	0	0	0	0	1	1
	Türkei_Antalya	0	0	0	0	0	0	1	0	1
	Türkei_Antalya_Portugal_Lissabon_Algarve	0	0	0	0	0	0	1	0	1
	Türkei_Datca	0	0	0	0	0	0	1	0	1
	Türkei_Konya	0	0	0	0	0	0	2	0	2
	Türkei_Side	0	0	0	0	0	0	3	0	3
	Ungarn	0	0	0	0	0	0	1	0	1
	Venedig_Rimini	0	1	0	0	0	0	0	0	1
	Venerien_Chioggia	0	1	0	0	0	0	0	0	1
	Verona	0	3	0	0	0	0	0	0	3
	Verona_Cavallino	0	1	0	0	0	0	0	0	1
	Viserba_Rimini	0	1	0	0	0	0	0	0	1
	Vrsar_Kroatein	0	0	1	0	0	0	0	0	1
	Zypern_Ayia_Napa	0	0	0	0	0	0	1	0	1
Total		411	303	93	40	36	11	28	66	988
